# Targeted killing of TNFR2-expressing tumor cells and T_regs_ by TNFR2 antagonistic antibodies in advanced Sézary syndrome

**DOI:** 10.1038/s41375-018-0292-9

**Published:** 2018-10-24

**Authors:** H Torrey, M Khodadoust, L Tran, D Baum, A Defusco, Y H Kim, D L Faustman

**Affiliations:** 1 Massachusetts General Hospital and Harvard Medical School, Building 149, 13th Street, Rm 3602, 02129 Boston, MA USA; 2 Stanford University School of Medicine/Cancer Institute, 94305 Palo Alto, CA USA

**Keywords:** Translational research, Cancer microenvironment

## Abstract

Sézary syndrome (SS) is a rare form of cutaneous T-cell lymphoma often refractory to treatment. SS is defined as adenopathy, erythroderma with high numbers of atypical T cells. This offers an opportunity for new interventions and perhaps antibody-based therapeutic by virtue of its high expression of the TNFR2 oncogene on the tumor cells and on T-regulatory cells (T_regs_). Potent human-directed TNFR2 antagonistic antibodies have been created that preferentially target the TNFR2 oncogene and tumor-infiltrating TNFR2^+^ T_regs_. Here we test the therapeutic potential of TNFR2 antagonists on freshly isolated lymphocytes from patients with Stage IVA SS and from healthy controls. SS patients were on a variety of end-stage multi-drug therapies. Baseline burden T_reg_/T effector (T_eff_) ratios and the responsiveness of tumor and infiltrating T_regs_ to TNFR2 antibody killing was studied. We show dose-escalating concentrations of a dominant TNFR2 antagonistic antibody killed TNFR2^+^ SS tumor cells and thus restored CD26^−^ subpopulations of lymphocyte cell numbers to normal. The abundant TNFR2^+^ T_regs_ of SS subjects are also killed with TNFR2 antagonism. Beneficial and rapid expansion of T_eff_ was observed. The combination of T_reg_ inhibition and T_eff_ expansion brought the high T_reg_/T_eff_ ratio to normal. Our findings suggest a marked responsiveness of SS tumor cells and T_regs_, to targeting with TNFR2 antagonistic antibodies. These results show TNFR2 antibodies are potent and efficacious in vitro.

## Introduction

Sezary Syndrome is a rare form of cutaneous T cell lymphoma. SS is considered a late stage and aggressive form lymphoma with a poor prognosis. SS subjects ave significant blood involvement with malignant T cells known as Sézary cells (SC) [1, 2]. Effective treatments for CTCL are limited and most forms of immunotherapy have shown minimal effectiveness. SS offers a special opportunity for an antibody-based therapeutic intervention because of its high expression of the newly characterized oncogene tumor necrosis factor receptor 2 (TNFR2) on tumor cells [3], as well as on T_reg_ cells in the tumor microenvironment [4–9]. In SS the significant blood involvement allows direct blood sampling of tumor cells and the tumor microenvironment [3].

Recent research finds that TNFR2 is expressed directly on tumor cells as an oncogene conferring preferential growth [10]. This is particularly well-depicted in SS where point mutations and genomic gains of TNFR2 (TNFRSF1B) lead to enhanced NFκB signaling for cell expansion and growth [3]. In non-tumor settings TNFR2 is the bi-directional switch for either T_reg_ expansion or T_reg_ contraction with limited systemic expression [11]. In SS alterations by point mutations or gain mutations suggest a potential role of oncogenic TNFR2 signaling and increased TNFRSF1B transcript mRNA with expanded expression onto the tumor cell themselves. Furthermore these SS mutations correlate with worse outcomes. Relatedly, the TNFR2 oncogene expression on ovarian tumor cells render them susceptible to death by TNFR2 antagonism: TNFR2 antagonistic antibodies in vitro directly kill TNFR2-expressing ovarian cancer cell lines, kill the tumor-associated TNFR2^+^ T_regs_, and proliferate the beneficial T effectors (T_effs_) [12, 13]. The TNFR2 oncogene is now expressed on at least 25 tumor types [14].

T_reg_ removal or inactivation in cancer is considered part of an essential strategy to remove or diminish host-generated immune suppression [15]. T_regs_ are a host-derived cell that suppresses the immune response and hampers host recognition of the cancer [16, 17]. Therefore, a targeted approach to remove or inactive host T_regs_ might better control cancer. While T_reg_ deficiency could put an individual at risk of developing autoimmune disease [18] or graft-versus-host disease (GvHD) [19], selective or transient deletion of the most suppressive T_regs_ in only the tumor microenvironment may provide optimal conditions for antitumor immune response [20]. Finding restricted or exclusive receptors specific to T_regs_ has been challenging [21–23]. Many surface receptor of T_regs_ are also expressed diffusely in the immune system, with TNFR2 being a prominent exception with highest density in the tumor microenvironment [13]. The TNFR2 receptor is a member of the TNF superfamily receptors that are composed of over 29 receptors. The expression of TNFR2 is limited: while most TNF superfamily member receptors are expressed on all lymphoid and sometimes all parenchymal cells, TNFR2 expression is restricted to a subpopulation of potent T_regs_, myeloid suppressor cells, and developing neurons [24, 25]. Research in primates finds that TNFR2-specific ligands have minimal in vivo systemic toxicity [26], most likely because of TNFR2’s restricted cellular distribution. Indeed unlike many other broadly expressed T_reg_ targets, TNFR2^−^^/^^−^ mice have no signs of autoimmunity. This combined with unique TNFR2 antagonistic antibodies that can only kill rapidly proliferating TNFR2-expressing cells creates a setting where tumor microenviroment T_reg_ targeting may be possible [13]. This combined with the limited natural expression of TNFR2 makes it an ideal target for a possible safer immunotherapy.

Naturally occurring T_regs_ have inducible TNFR2 expression with a tenfold higher density than TNFR1, the other most closely related receptor of the TNFR superfamily. TNFR2 receptor lacks an intracellular death domain and thus is a cell growth pathway linked to NFκB and thus cell growth through this known proliferative pathway. TNFR2 is preferentially expressed on T_regs_ and is a functional receptor—indeed, the master switch—for T_reg_ survival in humans and mice [11]. T_regs_ die with TNFR2 blockade or lack of stimulation in development or adulthood. Therefore, the TNFR2 surface protein is not merely an identifier of potent T_regs_, but is the central control site for T_reg_ survival.

T_regs_ in both mouse and humans that express the TNFR2 receptor are potently suppressive and are the predominant infiltrating cells found in human and murine tumors [4–6, 8, 9, 27]. In some human cancers, the expression of TNFR2 on infiltrating T_regs_ is estimated to be 100 times higher than on circulating T_regs_ in control subjects. In other forms of human cancer, the overall abundance of TNFR2^+^ T_regs_ is higher than in peripheral blood [9, 28]. In human cancers, the most suppressive T_regs_ express excess TNFR2 receptors on their surface and exert very potent host immunosuppressive effects [9, 28]. Agonism of the TNFR2 receptor results in membrane cleavage, generating soluble TNFR2 (sTNFR2), a serum marker diagnostic of poor cancer outcomes [29–33]. These features make TNFR2 an advantageous molecular target on T_regs_ and form the basis for T_reg_ inactivation approach using human-directed antibodies to TNFR2 for cancer therapies [10].

This in vitro study examines end-stage SS subjects for susceptibility of their lymphocytes and cancer cells in culture to a TNFR2 antagonistic antibody. Subjects were receiving a variety of treatments with advanced disease (Stage IVA). With the discovery of TNFR2 signaling pathway polymorphisms and mutations in SS and long prior course of diverse therapeutic interventions, it is vital to show that TNFR2 antagonistic antibodies have the capacity to kill TNFR2^+^ T_regs_ and TNFR2 oncogene-expressing tumor cells.

## Results

### End-stage Sézary syndrome patients express higher TNFR2 in tumor-residing CD26^−^ cells and T_regs_

**Fig. 1:**
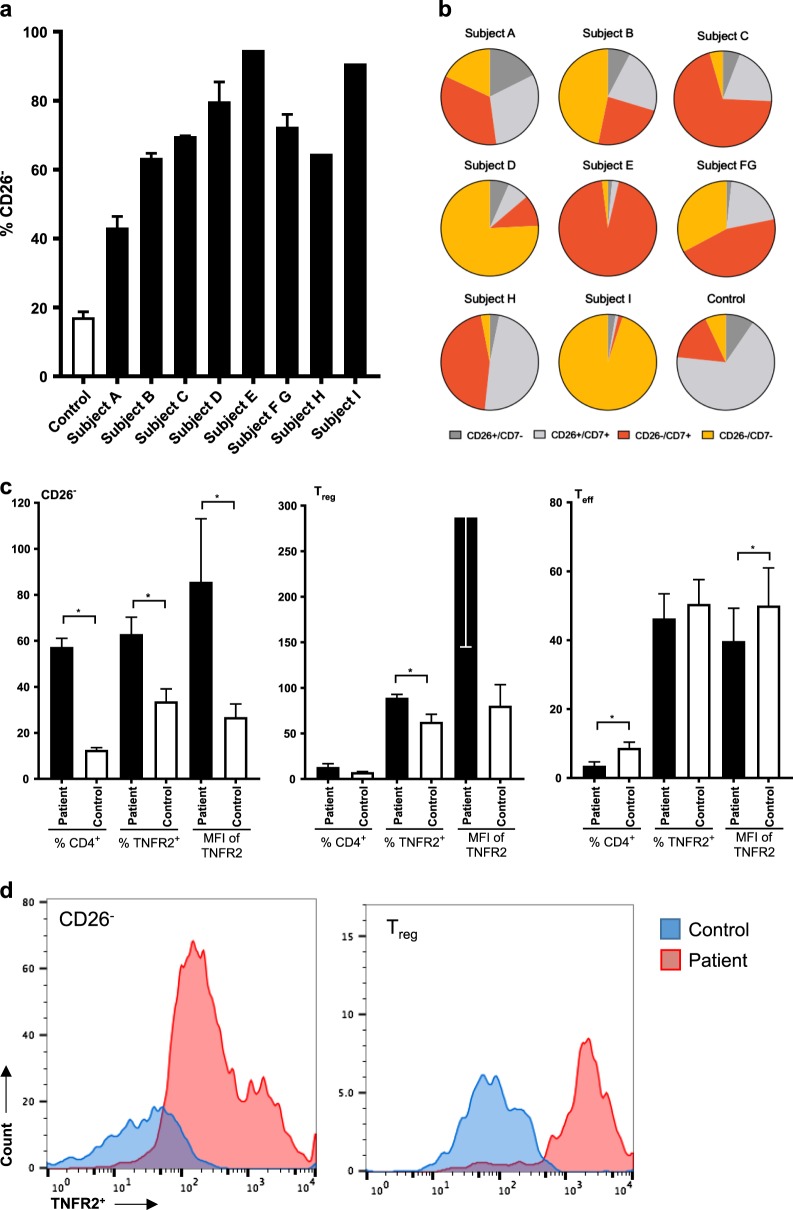
Sézary syndrome patients exhibit characteristically high levels of CD26^−^ cells and T_regs_ with increased TNFR2 expression. **a** Percentage of CD26^−^ cells from freshly isolated peripheral human CD4^+^ cells of Sézary syndrome patients and healthy controls (*n* = 1 Subjects E, H, I; *n* *=* 2 Subjects A, D, C; *n* = 3 Subjects B, FG, and *n* = 10 Controls). **b** Proportion of CD26^+/^^−^ and CD7^+/^^−^ lymphocytes. **c**. Proportion of lymphocyte populations (CD26^−^, T_reg_, and T_eff_) and TNFR2 expression of all patients except samples from subjects with tumor cells > 90% of CD4+ (Subjects E, I) and controls (*n* = 11 Patients, *n* = 11 Controls). **d** Individual histograms showing the massive amounts of TNFR2 expression on either tumor containing CD26^−^ cells or T_reg_, cells in a characteristic Sezary syndrome subject (pink) compared to a control subject (blue). Data are mean ± SEM, underlined asterisks indicate significant difference between patient and controls determined by *Z* test (95% CI)

We initially characterized CD4^+^ cells from advanced SS patients (Supplementary Table S1). Abnormally low or no surface expression of CD26 on CD4^+^ T cells is a characteristic of the malignant cells of SS patients and a useful diagnostic marker of disease [34]. In all end-stage SS subjects, the proportion of CD26^−^ CD4^+^ cells ranged from 40 to over 90% whereas the proportion in healthy controls was <20% (Fig. 1a). All subjects were in Stage IVA of their disease. The lack of CD7 on CD4^+^ cells is another indicator of disease progression [35]. These same subjects were found to exhibit varying degrees of CD7 expression, indicating varying disease progression (Fig. 1b). Adding to the diversity, each subject was on a unique treatment regimen and samples were taken at various stages of treatment (Supplementary Tables S1 and S2).

Next, we assessed the level of TNFR2 expression. As expected, we found a significantly higher proportion of TNFR2^+^ CD26^−^ and TNFR2^+^ T_regs_ in SS patients than controls (*Z* test, 95% CI) (Fig. 1c). In addition to the greater proportion of TNFR2^+^ cells, others have found higher TNFR2 transcript levels in patient tumor samples [36]. Indeed, we found that the mean florescence intensity (MFI) of TNFR2 on CD26^−^ and T_regs_ was also higher in patients, indicating higher receptor density (Fig. 1c). In contrast, with T_eff_, the proportion of TNFR2^+^ cells and the TNFR2 MFI was significantly lower in patients than healthy controls (Fig. 1c). In one patient where malignant clone-specific TCR Vb was determinable (Subject E), CD26^−^SC were enriched in the Vb-positive subset and the MFI of TNFR2 was higher (Supplementary File S2a). In another patient (Subject C), TNFR2^+^ CD26^−^ SC of clone-specific Vb-positive cells were more susceptible to the effect of TNFR2 antagonism than non-clonal cells (Supplementary File S2b). A set of representative flow cytometry histogram of the MRI of TNFR2 on tumor cells and on T_reg_ cells compared to control cells shows on a log scale the massive expression of TNFR2 oncogene on these two cells types in this cancer during advanced disease (Fig. 1d). Taken together, these results support abnormally high CD4^+^ CD26^−^ phenotype, demonstrate variability in the CD7 profile, and reveal significant differences in level of TNFR2 expression in SS patients compared to controls both with high expression on the tumor cells themselves and on the associated tumor-associated T_regs_. They also suggest tumor-specific expression and possible merit for looking for sensitivity of the TNFR2 target to targeted immunotherapy.

### A dominant TNFR2 antagonist antibody eliminates TNFR2^+^ CD26^−^ cells of Sézary syndrome patients

**Fig. 2:**
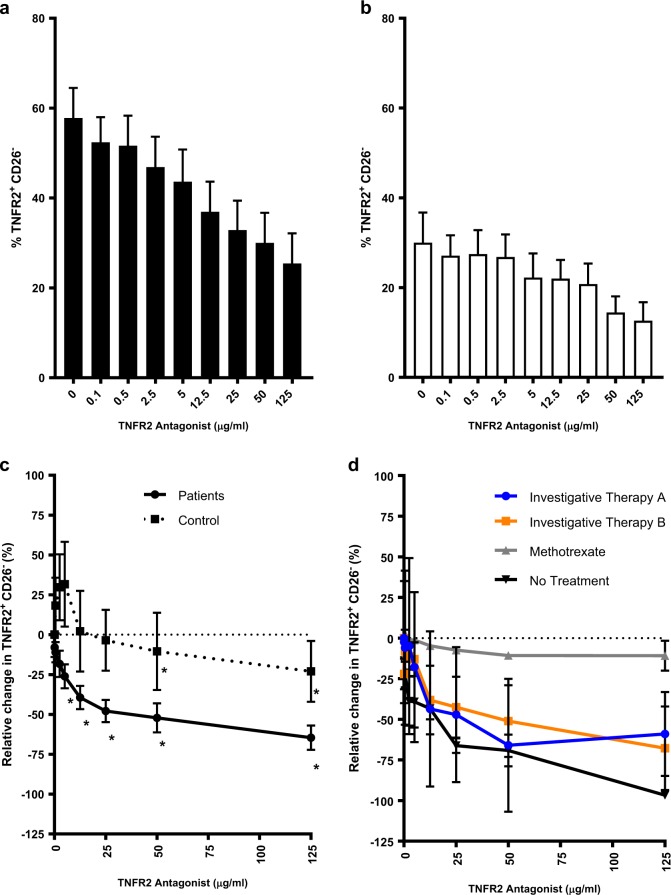
TNRF2^+^ CD26^−^ cells are reduced in response to treatment with TNFR2 antagonist. **a** Proportion of TNRF2^+^ CD26^−^ cells from Sézary syndrome patients (*n* = 15) after treatment with IL-2 (200 U/ml) and TNFR2 antagonist (0–125 µg/ml) for 48 to 72 h. **b** Proportion of TNRF2^+^ CD26^−^ cells from healthy controls (*n* = 11) treated as described in **a**. **c** Relative change in TNFR2^+^ CD26^−^ cells in patients and controls as described in **a**. **d** Relative change in TNFR2^+^ CD26^−^ cells in patients on various treatment regimens (Investigative Therapy A (*n* = 4), Investigative Therapy B (*n* = 3), methotrexate (*n* = 2), Control (*n* = 11). Data are mean ± SEM and asterisks indicates significant difference from baseline (TNFR2 antagonist 0 µg/ml (**a, b**) or 0.1 µg/ml (**c**)) (*t* test *p* < 0.05), and underlined asterisks indicate significant difference in patients versus controls (*Z* test 95% CI)

We previously reported the elimination of TNFR2-expressing T_regs_ and TNFR2-expressing ovarian cancer cells in a dose-dependent manner by dominant TNFR2 antagonistic antibodies [13]. Here we demonstrate that tumor-residing TNFR2^**+**^ CD26^−^ are also susceptible to the inhibitory effects of one of the TNFR2 antagonists used in the ovarian culture study. Even in short assays (48 to 72 h), the proportion of TNFR2^+^ CD26^−^ cells was significantly reduced (*t* test, *p* < 0.05 for TNFR2 antagonist ≥ 12.5 µg/ml) in SS patient samples samples; Fig. 2a and Supplementary File S3a). In healthy controls, even though the baseline proportion of CD26^−^ and TNFR2^+^ CD26^−^ cells was considerably lower than in patients (Fig. 1c; *Z* test, 95% CI), a significant reduction (*t* test, *p* < 0.05 for TNFR2 antagonist ≥ 50 µg/ml) in TNFR2^**+**^ CD26^−^ was observed (Fig. 2b). Importantly, after normalizing the data we found that the relative change in TNFR2^**+**^ CD26^−^cells was significantly greater (*Z* test, 95% CI) at a tenfold lower dose in patients (5 µg/ml) than controls (50 µg/ml; Fig. 2c and Supplementary File S3b). This suggests that tumor-residing CD26^−^ cells of SS patients are more sensitive to the action of the TNFR2 antagonist than CD26^−^ cells of healthy controls. This may be due to faster turnover of the TNFR2 target on proliferating cancer cells. Importantly, we confirmed that the reduction in the proportion of CD26^−^ cells, due to TNFR2 antagonist treatment, equates to a reduction in total CD26^−^ cell number (Supplementary File S4a-d).

An important consideration of combination cancer therapy is the possibility that one type of therapy modulates the efficacy of another type of therapy. To assess whether SS patients’ treatment regimens affect the in vitro efficacy of TNFR2 antagonist, we analyzed patient samples by treatment type. Interestingly, samples from treatment-naive patients or those on Investigative Therapy A or B were significantly more susceptible (*t* test, *p* < 0.05) to the TNFR2 antagonist (≥50 µg/ml) than samples from a methotrexate (MTX)-treated patient (Fig. 2d). While it is possible that the neoplastic lymphocytes obtained from the MTX patient sample were less responsive to the TNFR2 antagonist than other patients, the findings are consistent with our previous observation that inhibition of DNA replication can interfere with efficacy of the antibody [13]. The mechanism of action of the TNFR2 antagonist antibodies, based on structural biology observations, is the capture of newly synthesized surface TNFR2 on rapidly proliferating cells. This capture makes TNFR2 an anti-parallel dimers that can inhibit cell growth and kills the cells by preventing of signaling trimmers [12, 13].

**Fig. 3:**
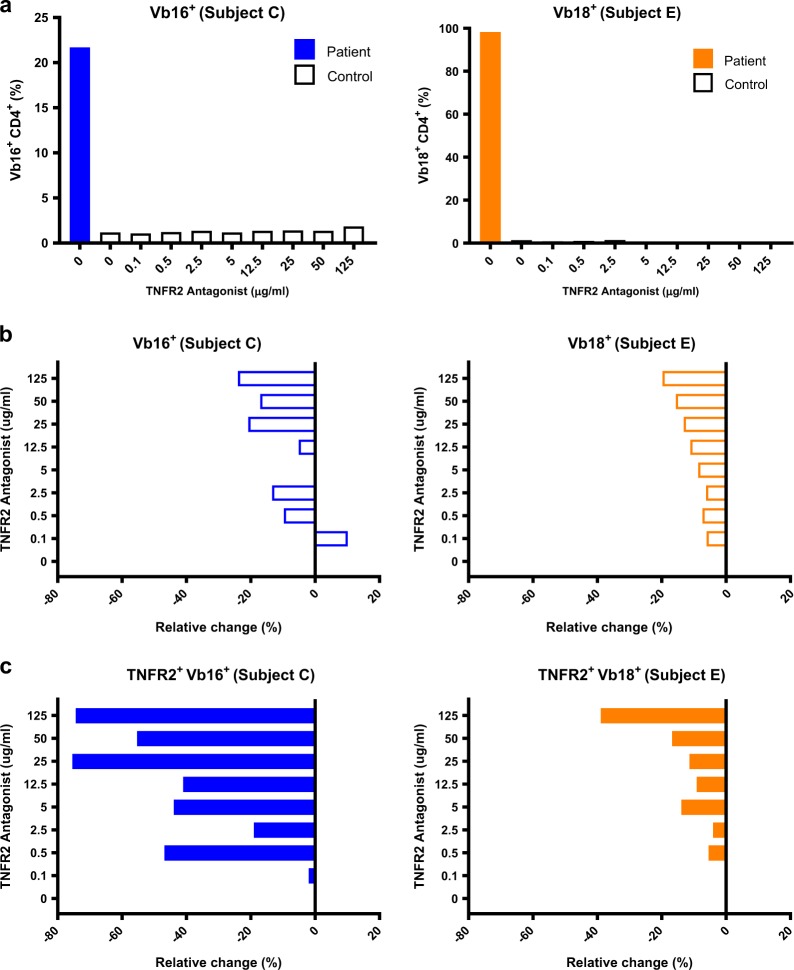
TCR Vbeta clonal CD4+ cells of Sézary syndrome patients are inhibited in a dose-dependent manner by treatment with the TNFR2 antagonist antibody. a. Proportion of CD4+ TCR Vb-specific cells in patient versus control for Subject C (left) and Subject E (right). b. Relative change in the proportion of CD4+ Vb-specific clonal cells for Subject C (left) and Subject E (right). c. Relative change in the proportion of TNFR2+ Vb-specific cells for Subject C (left) and Subject E (Right) (bottom). Data are from a single representative experiment

In two patients (Subject C, E), clonal malignant T cells were determined by TCR Vb subtyping. To assess the effect of the TNFR2 antagonistic antibody on the clonal population, we isolated these T cells for direct examination. In both cases, treatment with the TNFR2 antagonist led to elimination of the clonal tumor cell populations (Fig. 3a, b and Supplementary File S5 a-d). As expected, the inhibitory effect of the antagonistic antibody was amplified for the subset expressing high levels of TNFR2 (Fig. 3b, c). These results demonstrate direct killing of tumor-residing cells by the TNFR2 antagonist.

### TNFR2 antibody inhibits T_regs_ and enables T_eff_ proliferation

**Fig. 4:**
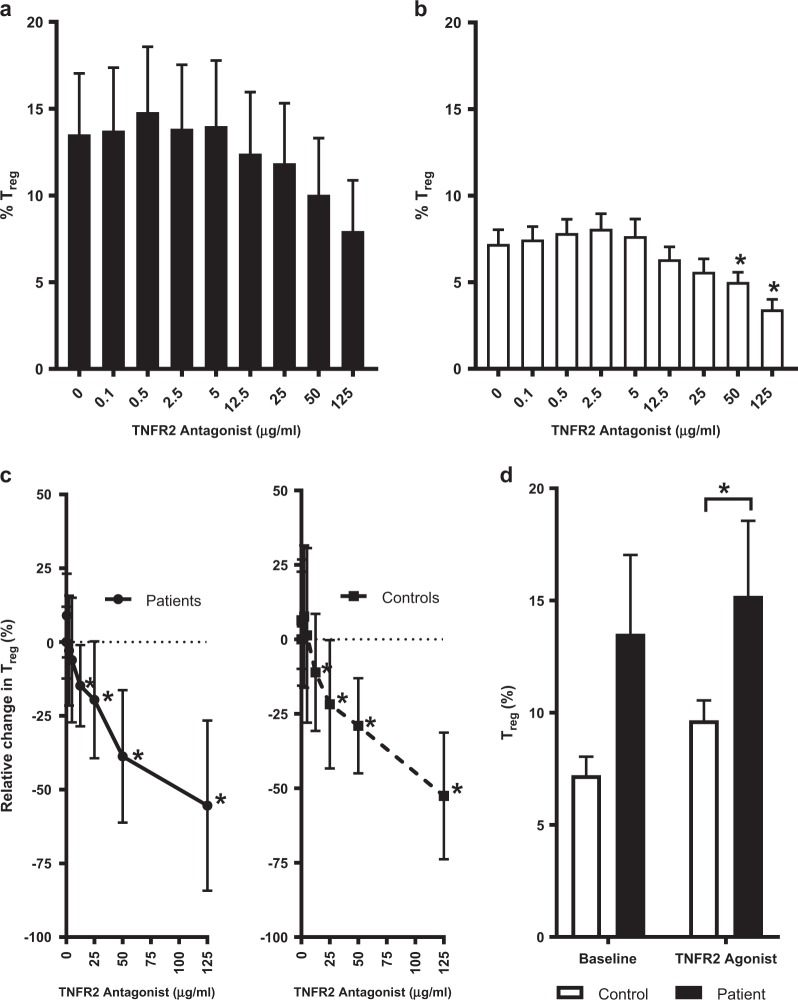
TNFR2 antagonist inhibits Tregs in Sézary syndrome and healthy control CD4+ cell culture. a. Proportion of Treg cells from patients (n = 15) after incubation of freshly isolated CD4+ cells with IL-2 and TNFR2 antagonist (0–125 µg/ml) for 48–72 hrs. b. Proportion of Treg cells from controls (n = 11) as described in (a). c. Relative change in Treg of patients and controls after culture with TNFR2 antagonist as described in (a). d. Comparison of baseline proportions of Treg from patients and controls treated with IL-2 alone or with TNFR2 agonist (12.5 µg/ml). Data are mean ± SEM, stars (*) indicate significant difference (T-test p<0.05) from baseline (TNFR2 antagonist 0 µg/ml (B) or 0.1 µg/ml (C)), and underlined stars (*) indicate significant difference in patients versus controls (Z-test 95%CI)

TNFR2^+^ T_regs_ collected from the tumor microenvironment are known to be highly immunosuppressive and reducing their presence is a major objective in cancer immunotherapy. Therefore, in addition to direct killing of tumor-residing T cells, we sought to assess whether T_regs_ from peripheral blood of SS patients would also be targeted by the TNFR2 antagonist. Indeed, pooled data of T_regs_ from SS patients demonstrated susceptibility to dose-dependent killing by the TNFR2 antagonist (Fig. 4a). While all patient samples were individually analyzed and responded as expected (Supplementary File S6a), it is important to note that Subjects E and I were removed from the pooled data due to very low numbers of T_regs_. The paucity of T_regs_ in these subjects was likely due to very high tumor-residing CD26^−^ cell populations (Fig. 1a) or treatment regimens targeting T_regs_ (Investigative Therapy B; Supplementary Tables S1 and S2). We assessed T_regs_ from healthy controls and observed the expected dose-dependent elimination by the TNFR2 antagonist (Fig. 4b).

Previously we reported that T_regs_ from the ovarian cancer tumor microenvironment were more susceptible to the effect of the TNFR2 antagonist than T_regs_ from healthy donors [13]. In this study, we predicted that T_regs_ from peripheral blood of SS patients would also be more susceptible than those of healthy controls. Normalized comparison of pooled data showed that both patients and controls exhibited significant reduction in T_regs_ from baseline at TNFR2 concentration ≥ 12.5 µg/ml (Fig. 4c and Supplementary File S6b). Thus, there was no significant difference in the degree of elimination of T_regs_. However, we did find that T_regs_ from peripheral blood of SS patients had a significantly greater (*p* < 0.05) response to treatment with a TNFR2 agonist than healthy controls (Fig. 4d). This data supports our observations with T_regs_ from ovarian cancer ascites fluid and further suggests that T_regs_ of the tumor microenvironment in SS are not only expressing very high levels of TNFR2 but presumably are also rapidly proliferating from the overabundance of this growth receptor. Also it should be mentioned that the ovarian cancer study used treatment-naive subjects just diagnosed with ovarian cancer, and this study used advanced SS subjects on a range of salvage therapies some of which might work to decrease in vivo T_regs_ cell numbers thus accounting for the more dramatic T_regs_ effect of TNFR2 antagonism.

**Fig. 5:**
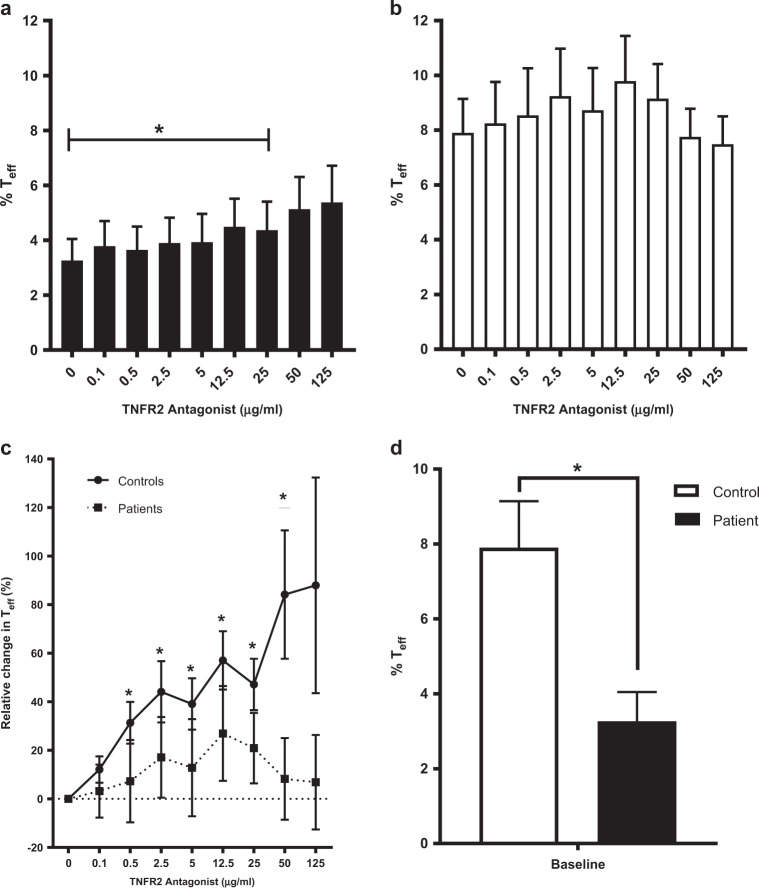
TNFR2 antagonist enables continuous dose-dependent T_eff_ expansion in Sézary syndrome patient samples. **a** Proportion of T_eff_ cells from patients (*n* = 18 samples) after incubation of freshly isolated CD4^+^ cells with IL-2 and TNFR2 antagonist (0–125 µg/ml) for 48–72 h. **b** Proportion of T_eff_ cells from controls (*n* = 11) as described in **a**. **c** Relative change in T_eff_ of patients and controls after culture with TNFR2 antagonist as described in **a**. **d** Comparison of baseline proportions of T_eff_ from patients and controls. Data are mean ± SEM, asterisks indicate significant difference from baseline (TNFR2 antagonist 0.1 µg/ml; *t* test *p* < 0.05), and underlined asterisks indicate significant difference in patients versus controls (*Z* test 95%CI)

In the absence of suppressive T_regs,_ T_eff_ cells proliferate due to the removal of this cell mediated suppression. We therefore predicted, with the reduction in T_regs_ by TNFR2 antagonist treatment, a corresponding expansion of T_eff_. As expected, we observed a dose-dependent proliferation of T_eff_ at low concentrations of TNFR2 antagonist (0.1–12.5 µg/ml) in both SS patients and controls (Fig. 5a, b and Supplementary File S7a). T_eff_ proliferation continued to increase with higher doses of the antibody (25–125 µg/ml) in SS patients whereas it tapered off at high doses of the antibody in controls (Fig. 5c and Supplementary File S7b). Interestingly, baseline levels of T_eff_ were significantly lower in the patients (Fig. 5d) and were only restored to normal levels (baseline of healthy control) in the presence of high doses of the TNFR2 antagonist (50–125 µg/ml).

### Ratio of T_reg_/T_eff_ is corrected by TNFR2 antagonist regardless of patient treatment history or treatment stage

**Fig. 6:**
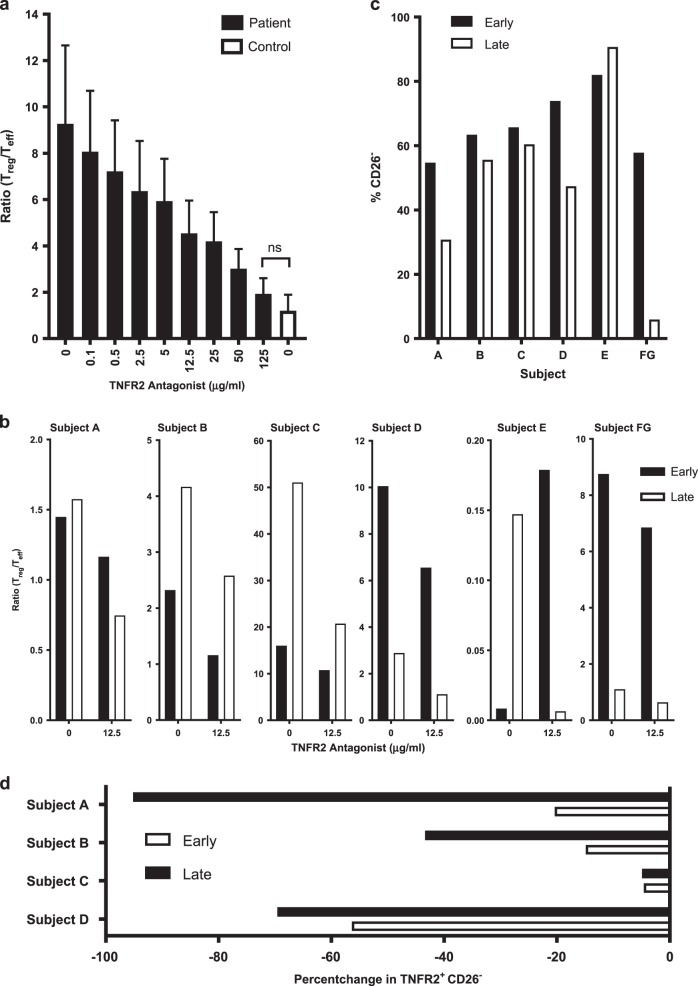
TNFR2 antagonism corrects T_reg_/T_eff_ ratio and reduces TNFR2^+^ tumor cells in Sézary syndrome patient samples regardless of underlying therapy. **a** Ratio of T_reg_/T_eff_ in patients (*n* = 15 samples) and controls (*n* = 11 samples) after incubation of freshly isolated CD4^+^ cells with IL-2 and TNFR2 antagonist (0–125 µg/ml) for 48–72 h. **b** Ratio of T_reg_/T_eff_ after TNFR2 antagonist treatment (0 and 12.5 µg/ml) as describe in **a** of early and late longitudinal samples from patients on various clinical treatment regimens. **c** Proportion of CD26^−^ of early and late patient samples at baseline (TNFR2 0 µg/ml). **d** Relative change in proportion of TNFR2^+^ CD26^−^ in samples treated with TNFR2 antagonist (12.5 µg/ml). Data are mean ± SEM and ns indicates no significant difference between patients and controls (*t* test *p* < 0.05)

The ratio of T_reg_/T_eff_ is an indicator of the suppressive capacity of the immune system in the tumor microenvironment. With a dose-dependent decrease in the T_regs_ and concurrent proliferation of T_eff_, we find that the TNFR2 antagonist has the ability to correct the T_reg_/T_eff_ ratio of SS patient samples. Indeed, the T_reg_/T_eff_ ratio of patients (*n* = 15 samples) was brought down to the baseline level of healthy controls (*n* = 11 samples) after only 48 to 72 h culture with the TNFR2 antagonist (125 µg/ml; Fig. 6a). To investigate whether this antibody would work effectively at various stages of clinical treatment, for each patient we tested two longitudinal samples which we assigned a label of early or late (Table S2) depending on the date of sample collection. In each case except one (Subject E), treatment of either sample with the TNFR2 antagonist (12.5 µg/ml) resulted in an improvement of the T_reg_/T_eff_ ratio (Fig. 6b). The very low T_reg_/T_eff_ ratio in Subject E is likely skewed due to the very high proportion of CD26^−^ in this patient (Fig. 1a). We also find that in all cases except one (Subject E), the proportion of tumor-residing CD26^−^ decreases from early to late in the sample comparison (Fig. 6c). With Subject E, the longitudinal increase in CD26^−^ may be explained by the fact that the patient was taken off, and remained off, therapy prior to sample collection. Importantly, for all patients receiving continuous therapy, there was a notable decrease in the relative change in TNFR2^+^ tumor-residing CD26^−^ cells in the longitudinal sample analysis (Fig. 6d).

## Discussion

Our population of SS patients had advanced disease (Stage IVA) and were on concurrent, multi-drug therapy. This advanced disease state was evident by massive tumor burdens within the CD26^−^ lymphocytes in peripheral blood. Our in vitro study reveals that TNFR2 antibody antagonism, despite advanced disease and diverse treatment regimens was effective in directly killing TNFR2^+^ tumor cells, eliminating high numbers of TNFR2^+^ T_regs_, permitting the expansion of T_eff_, and restoring T_regs_/T_eff_ ratios. This data suggests TNFR2 antagonism could potentially offer a clinical benefit once developed for in vivo therapy.

The mechanism by which TNFR2 antagonist kills TNFR2^+^ tumor cells and TNFR2^+^ T_regs_ is likely by blocking activation of NFkB, a transcription factor necessary for cell survival. The mechanism by which TNFR2 enables T_eff_ expansion is by lifting T_reg_ suppression [11].

Designing a dominant TNFR2 antagonistic antibody that can successfully exert effects in the presence of TNF has been difficult. This has also been true for antibody antagonists to other receptors of the TNF receptor superfamily. The past failures to create effective TNFR2 antagonistic antibodies have been largely due to the natural ligand, TNF, being a potent agonist that stabilizes the TNFR2 into a trimer with tight associations not dislodged by even high affinity antibodies. Thus the natural ligand—trimeric TNF, or trimeric membrane TNF—is nearly always dominant and when present result in agonism of TNFR2 and cell expansion. The TNFR2 antagonistic antibody used in this study is still effective in spite of high doses of TNF [13]. This dominant antagonism is possible by the selective capture of newly synthesized, non-assembled TNFR2 proteins on rapidly proliferating cells prior to trimerization with TNF. The newly formed structure, TNFR2 plus antagonistic antibody, is an assembly of newly synthesized TNFR2 as an anti-parallel dimer [10, 12]. This anti-parallel dimer blocks the TNF binding site and may also form a potent inhibitory signaling lattice. Dominant TNFR2 antagonistic antibodies also have specificity for the tumor-bearing cells and tumor-associated T_regs_ due to their rapid proliferation and thus surface supply of newly synthetized TNFR2 surface protein.

This brings us to the therapy being used in SS and its impact on TNFR2 antagonism effectiveness. This study suggests that dominant TNFR2 antibody antagonism effectiveness remains even in end-stage subjects. In metastatic melanoma it is known that both anti-CTLA4 and anti-PDL1 treatment failures are driven by a tumor microenvironment full of TNFR2^+^ T_regs_, suggesting that TNFR2 could be a similar escape pathway for the lost effectiveness on current therapies in SS [37]. There is one exception that will need to be repeated with further studies. As observed in Fig. 2d, if the SS subjects are on methotrexate, the TNFR2 antagonist-induced killing of SS tumor cells in culture appears to be blunted. It is possible that the neoplastic lymphocytes of this patient were inherently less susceptible. However, further support is provided by observations that TNFR2^+^ CD26^−^ cells of patients were more susceptible than those healthy controls, and that those of naive patients were more susceptible than those of treated patients, since all treatments for SS aim to slow down the proliferation of neoplastic lymphocytes. This was also previously reported in ovarian cancer subjects exposed to Mitomycin C, another anti-proliferative agent [13]. A diminished TNFR2 antagonistic antibody killing response against ovarian cancer or against tumor-associated T_regs_ appears to occur if the cell target is not rapidly proliferating. This clinical situation with SS is consistent with dominant TNFR2 antagonistic antibodies having the enhanced ability to target newly produced surface TNFR2 if the cancer cells are rapidly proliferating. If rapid proliferation is inhibited by concurrent therapy, our TNFR2 antagonist is less effective. It is known that the dominant antibody antagonism has such T_reg_ specificity since the antibody captures a unique newly synthesized anti-parallel TNFR2 dimer on the surface of rapidly proliferating cells [12, 38].

Genetic studies in Sezary Syndrome have identified many alterations in the key T cell signaling pathways for T cell expansion. Two genetic studies similarly identify TNFR2 mutations and all studies have observed involvement of the downstream NFkB proteins to surface TNFR2 cell signaling [39–41].

In summary, TNFR2 is an oncogene expressed on some cancers, such as SS, and a cell surface protein found in abundance on tumor-associated T_regs_. In this study, we show that dominant TNFR2 antagonistic antibody can kill completely the abundant T_regs_ of SS, directly kill the tumor cells and this unleashes rapid expansion of T_eff_ cell numbers to corrected T_reg_/T_eff_ ratios like controls. A recent limitation of moving TNFR2 antagonistic therapies into human trials has been the lack of a mouse surrogate antibodies for murine tumor studies. This issue has been recently solved with new data using a mouse-directed TNFR2 antagonistic antibody that shows efficacy in treating two murine models of cancer [42]. Taken together, these findings support TNFR2 antagonism as a new multi-pronged approach in difficult-to-treat end-stage SS with implications for numerous other TNFR2-expressing tumors. Indeed recent data similarly shows TNF signaling in the malignant cell of myelofibrosis proliferate and expand preferentially through the TNFR2 receptor and linked pathway of clonal expansion [38]

## Materials and methods

### Human subjects

Human blood samples were collected from SS patients (*n* = 18 from 8 SS subjects) and healthy controls (*n* = 11 samples from 11 subjects) according to a human studies protocol approved by the Massachusetts General Hospital (MGH) Human Studies Committee (MGH-2001P001379) and Stanford University Human Studies Committee (IRB 5538 and IRB 13844). All the donors provided written informed consent. We also stipulated with our request for patient samples that the study drugs were not anti-mitotic drug since we know from published data that this alone will interfere with our effectiveness with TNFR2 antagonism that require rapidly proliferating cells.

Blood was collected into BD Vacutainer EDTA Tubes (BD Diagnostics) and processed within 24 h of phlebotomy. These human studies were approved by the MGH Human Studies Committee (MGH-2015P002489).

### Blood and cell culture

Fresh human blood was processed within 24 h of venipuncture. CD4^+^ cells were isolated using the Direct Human CD4^+^ T Cell Isolation Kit (Stemcell Technologies). Isolated CD4^+^ cells were re-suspended in RPMI GlutaMAX (Life Technologies) plus 10% FBS (Sigma-Aldrich) and 1% penicillin streptomycin (Life Technologies). Because isolated and cultured human T cells die without IL-2 in the media, all culture conditions in all experiments contained a low amount of IL-2 (200 U/ml) to prevent IL-2 withdrawal from influencing the data.

### Cell culture assays

For cell culture assays, freshly isolated CD4^+^ cells were seeded at a concentration of 0.2 × 10^6^ cells per well in 96-well round-bottom plates, treated with the TNFR2 antagonist or agonist, and incubated at 37 °C and 5% CO_2_ for 48 to 72 h. After incubation, cells were collected by centrifugation, washed once with 1× Hanks’ balanced salt solution (HBSS) (Invitrogen), and stained for FACS analysis.

### Reagents and flow cytometry

Monoclonal antibodies (mAbs) against human TNFR2 were produced internally as previously described [11]. Recombinant human TNF was purchased from Sigma-Aldrich and recombinant human IL-2 was purchased from Life Technologies. Cells were prepared for flow cytometry using Human T_regs_ Flow Kit (BioLegend) according to the manufacturer’s instructions. Fluorescently stained cells were re-suspended in 1× HBSS (Invitrogen) and analyzed using a BD FACS Calibur Flow Cytometer machine (Becton Dickinson). Antibodies used for FACS analysis of T_regs_ included AlexaFluor 488 anti-human FOXP3 (Clone 259D; BioLegend) for intracellular staining of FOXP3, phycoerythrin (PE) human CD25 (Clone BC96; BioLegend), PE/Cy7 human CD26 (Clone BA5b; BioLegend), and fluorescein isothiocyanate (FITC) human CD7 (Clone CD7-6B7; BioLegend) for cell surface staining of CD25, CD26, and CD7 respectively. Anti-human TNFR2/TNFRSF1B (MAB726; R&D Systems) was conjugated to allophycocyanin (APC) by Lightning-Link (Innova Biosciences) and used for assessing TNFR2 cell surface expression by FACS with FL4 (far red). T_reg_ populations were assessed by FACS with FL2 (red) versus FL1 (green) and defined as CD25hi and FoxP3^−^positive, T_eff_ populations were defined as CD25^hi^ and FoxP3^-^negative, CD26^−^ cells were defined as CD26^low^ (Supplementary File S1). To be able to best discern T_reg_ cells from Sezary cells that also express Foxp3, all flow cytometry gating utilized only CD4 T cells with high CD25, lacking CD127; this was done for identifying the most suppressive human T_reg_ cells (TNFR2+) and minimizing the contamination of possible SS cells [43].

T cell receptor (TCR) Vbeta (Vb) 16 clone cells were isolated from CD4+ cell suspensions by labeling with FITC conjugated TCR Vbeta 16 mAb (Beckman-Coulter) and extracting the FITC-labeled cells with EasySep^TM^ FITC Positive Selection Kit (Stemcell Technologies) and assessed by FACS with FL1 (green). TCR Vbeta18 clone cells were labeled with PE conjugated Vbeta 18 mAb (Beckman-Coulter) and assessed by FACS with FL2 (red). FACS data were processed using FlowJo software (version 10.1).

### Statistical analysis

Data analysis was performed by Student’s *t* test (unpaired, type 3) using Excel (Microsoft) or GraphPad Prism 5 software (GraphPad Software). Significance was determined by *P* < 0.05.

## Electronic supplementary material

Supplemental Tables, Figures, Legends

## References

[1] Swerdlow S. H., Campo E., Pileri S. A., Harris N. L., Stein H., Siebert R., Advani R., Ghielmini M., Salles G. A., Zelenetz A. D., Jaffe E. S. (2016). The 2016 revision of the World Health Organization classification of lymphoid neoplasms. Blood.

[2] Kim EJ, Hess S, Richardson SK, Newton S, Showe LC, Benoit BM (2005). Immunopathogenesis and therapy of cutaneous T cell lymphoma. J Clin Invest.

[3] Ungewickell A, Bhaduri A, Rios E, Reuter J, Lee CS, Mah A (2015). Genomic analysis of mycosis fungoides and Sezary syndrome identifies recurrent alterations in TNFR2. Nat Genet.

[4] Byrne WL, Mills KH, Lederer JA, O’Sullivan GC (2011). Targeting regulatory T cells in cancer. Cancer Res.

[5] Teng MW, Ritchie DS, Neeson P, Smyth MJ (2011). Biology and clinical observations of regulatory T cells in cancer immunology. Curr Top Microbiol Immunol.

[6] Facciabene A, Motz GT, Coukos G (2012). T-regulatory cells: key players in tumor immune escape and angiogenesis. Cancer Res.

[7] Chen X, Subleski JJ, Kopf H, Howard OM, Mannel DN, Oppenheim JJ (2008). Cutting edge: expression of TNFR2 defines a maximally suppressive subset of mouse CD4 + CD25 + FoxP3 + T regulatory cells: applicability to tumor-infiltrating T regulatory cells. J Immunol.

[8] Chen X, Subleski JJ, Hamano R, Howard OMZ, Wiltrout RH, Oppenheim JJ (2010). Co-expression of TNFR2 and CD25 identifies more of the functional CD4( + )FOXP3( + ) regulatory T cells in human peripheral blood. Eur J Immunol.

[9] Govindaraj C, Scalzo-Inguanti K, Madondo M, Hallo J, Flanagan K, Quinn M (2013). Impaired Th1 immunity in ovarian cancer patients is mediated by TNFR2 + Tregs within the tumor microenvironment. Clin Immunol.

[10] Vanamee ES, Faustman DL (2017). TNFR2: a novel target for cancer immunotherapy. Trends Mol Med.

[11] Okubo Y, Mera T, Wang L, Faustman DL (2013). Homogeneous expansion of human T-regulatory cells via tumor necrosis factor receptor 2. Sci Rep.

[12] Vanamee Éva S., Faustman Denise L. (2018). Structural principles of tumor necrosis factor superfamily signaling. Science Signaling.

[13] Torrey H, Butterworth J, Mera T, Okubo Y, Wang L, Baum D (2017). Targeting TNFR2 with antagonistic antibodies inhibits proliferation of ovarian cancer cells and tumor-associated Tregs. Sci Signal.

[14] Uhlen M, Bjorling E, Agaton C, Szigyarto CA, Amini B, Andersen E (2005). A human protein atlas for normal and cancer tissues based on antibody proteomics. Mol Cell Proteom.

[15] Drake CG, Lipson EJ, Brahmer JR (2014). Reply: Regulatory T cells-an important target for cancer immunotherapy. Nat Rev Clin Oncol.

[16] Scott AM, Wolchok JD, Old LJ (2012). Antibody therapy of cancer. Nat Rev Cancer.

[17] Wolf AM, Wolf D, Steurer M, Gastl G, Gunsilius E, Grubeck-Loebenstein B (2003). Increase of regulatory T cells in the peripheral blood of cancer patients. Clin Cancer Res.

[18] Zhang W, Sharma R, Ju ST, He XS, Tao Y, Tsuneyama K (2009). Deficiency in regulatory T cells results in development of antimitochondrial antibodies and autoimmune cholangitis. Hepatology.

[19] Li Q, Zhai Z, Xu X, Shen Y, Zhang A, Sun Z (2010). Decrease of CD4( + )CD25( + ) regulatory T cells and TGF-beta at early immune reconstitution is associated to the onset and severity of graft-versus-host disease following allogeneic haematogenesis stem cell transplantation. Leuk Res.

[20] Klages K, Mayer CT, Lahl K, Loddenkemper C, Teng MW, Ngiow SF (2010). Selective depletion of Foxp3 + regulatory T cells improves effective therapeutic vaccination against established melanoma. Cancer Res.

[21] Shimizu J, Yamazaki S, Sakaguchi S (1999). Induction of tumor immunity by removing CD25 + CD4 + T cells: a common basis between tumor immunity and autoimmunity. J Immunol.

[22] Sutmuller RP, van Duivenvoorde LM, van Elsas A, Schumacher TN, Wildenberg ME, Allison JP (2001). Synergism of cytotoxic T lymphocyte-associated antigen 4 blockade and depletion of CD25( + ) regulatory T cells in antitumor therapy reveals alternative pathways for suppression of autoreactive cytotoxic T lymphocyte responses. J Exp Med.

[23] Smyth MJ, Ngiow SF, Teng MW (2014). Targeting regulatory T cells in tumor immunotherapy. Immunol Cell Biol.

[24] Heinrich M, Burger D, Wang L, Tahhan G, Reinhold P, Zhao M (2015). TNFR1 and TNFR2 expression and induction on human Treg cells from type 1 diabetic subjects. Antibodies.

[25] Polz J, Remke A, Weber S, Schmidt D, Weber-Steffens D, Pietryga-Krieger A (2014). Myeloid suppressor cells require membrane TNFR2 expression for suppressive activity. Immun Inflamm Dis.

[26] Welborn MB, Van Zee K, Edwards PD, Pruitt JH, Kaibara A, Vauthey JN (1996). A human tumor necrosis factor p75 receptor agonist stimulates in vitro T cell proliferation but does not produce inflammation or shock in the baboon. J Exp Med.

[27] Chen X, Wu X, Zhou Q, Howard OM, Netea MG, Oppenheim JJ (2013). TNFR2 is critical for the stabilization of the CD4 + Foxp3 + regulatory T. cell phenotype in the inflammatory environment. J Immunol.

[28] Govindaraj C, Tan P, Walker P, Wei A, Spencer A, Plebanski M (2014). Reducing TNF receptor 2 + regulatory T cells via the combined action of azacitidine and the HDAC inhibitor, panobinostat for clinical benefit in acute myeloid leukemia patients. Clin Cancer Res.

[29] Tarhini AA, Lin Y, Yeku O, LaFramboise WA, Ashraf M, Sander C (2014). A four-marker signature of TNF-RII, TGF-alpha, TIMP-1 and CRP is prognostic of worse survival in high-risk surgically resected melanoma. J Transl Med.

[30] Heemann C, Kreuz M, Stoller I, Schoof N, von Bonin F, Ziepert M (2012). Circulating levels of TNF receptor II are prognostic for patients with peripheral T-cell non-Hodgkin lymphoma. Clin Cancer Res.

[31] Babic A, Shah SM, Song M, Wu K, Meyerhardt JA, Ogino S (2016). Soluble tumour necrosis factor receptor type II and survival in colorectal cancer. Br J Cancer.

[32] Warzocha K, Bienvenu J, Ribeiro P, Moullet I, Dumontet C, Neidhardt-Berard EM (1998). Plasma levels of tumour necrosis factor and its soluble receptors correlate with clinical features and outcome of Hodgkin’s disease patients. Br J Cancer.

[33] Dobrzycka B, Terlikowski SJ, Kowalczuk O, Kinalski M (2009). Circulating levels of TNF-alpha and its soluble receptors in the plasma of patients with epithelial ovarian cancer. Eur Cytokine Netw.

[34] Jones D, Dang NH, Duvic M, Washington LT, Huh YO (2001). Absence of CD26 expression is a useful marker for diagnosis of T-cell lymphoma in peripheral blood. Am J Clin Pathol.

[35] Rappl G, Abken H, Muche JM, Sterry W, Tilgen W, Andre S (2002). CD4 + CD7- leukemic T cells from patients with Sezary syndrome are protected from galectin-1-triggered T cell death. Leukemia.

[36] Lee CS, Ungewickell A, Bhaduri A, Qu K, Webster DE, Armstrong R (2012). Transcriptome sequencing in Sezary syndrome identifies Sezary cell and mycosis fungoides-associated lncRNAs and novel transcripts. Blood.

[37] Tirosh I, Izar B, Prakadan SM, Wadsworth MH, Treacy D, Trombetta JJ (2016). Dissecting the multicellular ecosystem of metastatic melanoma by single-cell RNA-seq. Science.

[38] Torrey H, Defusco A, Baum D, Rhabar Z, Khodadoust M, Kim YH et al. Novel treatment of cutaneous T cell lymphoma: targetting TNFR2 an oncogene and marker of potent Treg. Cancer Research 78 (13 Suppl):1790–1790.

[39] Wang L, Xiao N (2015). Genomic profiling of Sezary Syndrome identifeis alternations of key T-cell singaling and differentiation genes. Nat Genet.

[40] da Silva Almeida A, Abate F, Khiabanian H (2015). The mutational landscape of cutaneous T cell lymphoma and Sezary syndrome. Nat Genet.

[41] Choi J, Goh G, Walradt T (2015). Genomic landscape of cutaneous T cell lymphoma. Nat Genet.

[42] Ni X, Jorgensen JL, Goswami M, Challagundla P, Decker WK, Kim YH (2015). Reduction of regulatory T cells by Mogamulizumab, a defucosylated anti-CC chemokine receptor 4 antibody, in patients with aggressive/refractory mycosis fungoides and Sezary syndrome. Clin Cancer Res.

[43] Nie Y, He J, Shirota H, Trivett AL, Yang, Klinman DM (2018). Blockade of TNFR2 signaling enhances the immunotherapeutic effect of CpG ODN in a mouse model of colon cancer. Sci Signal.

