# Supplementary material for: Targeted killing of TNFR2-expressing tumor cells and Tregs by TNFR2 antagonistic antibodies in advanced Sézary syndrome
Source: Leukemia. 2018 Oct 24;33(5):1206–18. doi: 10.1038/s41375-018-0292-9 (PMC6756055; doi:10.1038/s41375-018-0292-9)
Supplement: Supplementary file 1 — Supplemental Tables, Figures, Legends [file 41375_2018_292_MOESM1_ESM.pptx]

## Slide 1
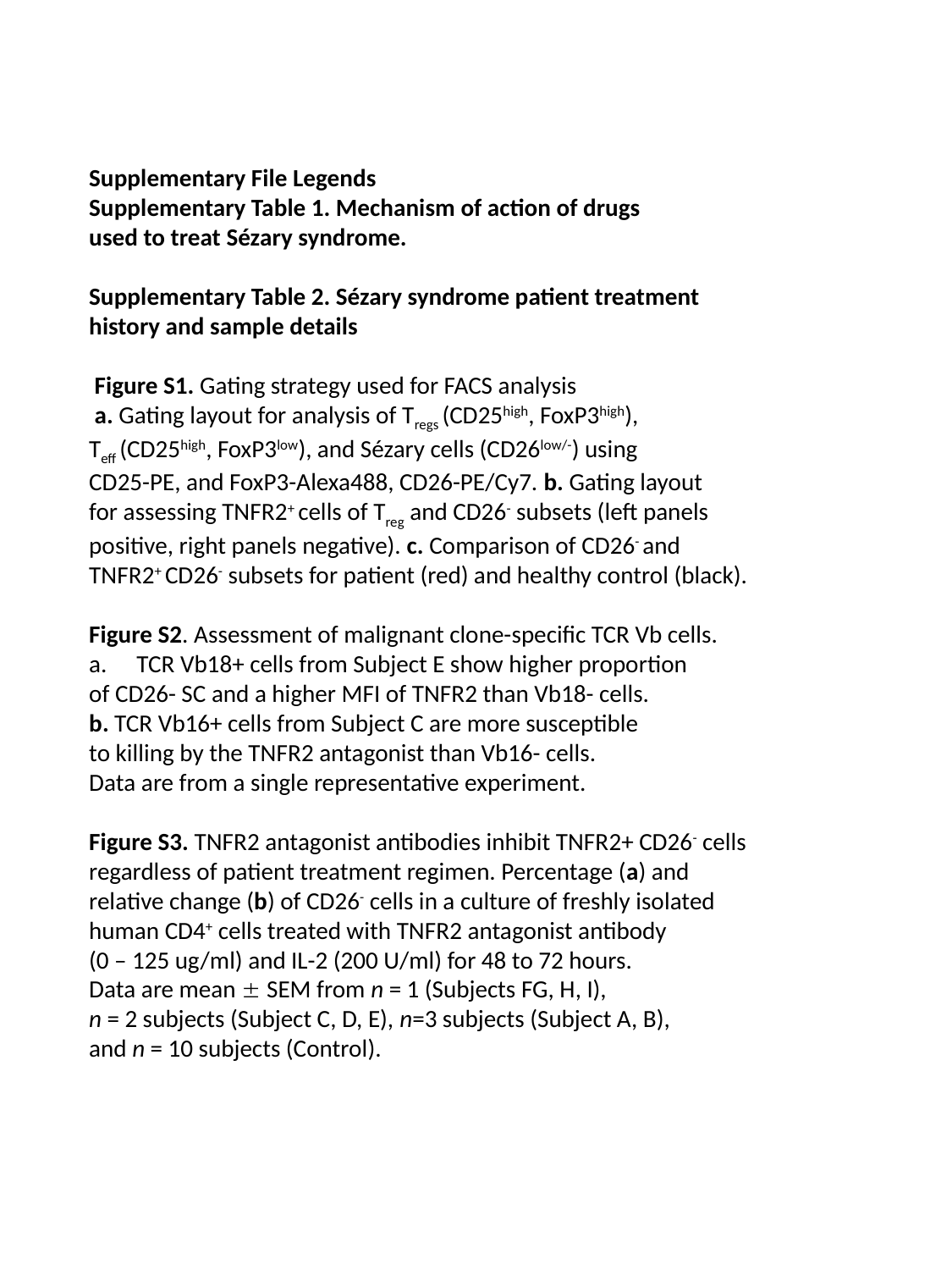

Supplementary File Legends
Supplementary Table 1. Mechanism of action of drugs
used to treat Sézary syndrome.
Supplementary Table 2. Sézary syndrome patient treatment
history and sample details
 Figure S1. Gating strategy used for FACS analysis
 a. Gating layout for analysis of Tregs (CD25high, FoxP3high),
Teff (CD25high, FoxP3low), and Sézary cells (CD26low/-) using
CD25-PE, and FoxP3-Alexa488, CD26-PE/Cy7. b. Gating layout
for assessing TNFR2+ cells of Treg and CD26- subsets (left panels
positive, right panels negative). c. Comparison of CD26- and
TNFR2+ CD26- subsets for patient (red) and healthy control (black).
Figure S2. Assessment of malignant clone-specific TCR Vb cells.
TCR Vb18+ cells from Subject E show higher proportion
of CD26- SC and a higher MFI of TNFR2 than Vb18- cells.
b. TCR Vb16+ cells from Subject C are more susceptible
to killing by the TNFR2 antagonist than Vb16- cells.
Data are from a single representative experiment.
Figure S3. TNFR2 antagonist antibodies inhibit TNFR2+ CD26- cells
regardless of patient treatment regimen. Percentage (a) and
relative change (b) of CD26- cells in a culture of freshly isolated
human CD4+ cells treated with TNFR2 antagonist antibody
(0 – 125 ug/ml) and IL-2 (200 U/ml) for 48 to 72 hours.
Data are mean ± SEM from n­ = 1 (Subjects FG, H, I),
n ­= 2 subjects (Subject C, D, E), n=3 subjects (Subject A, B),
and n = 10 subjects (Control).

## Slide 2
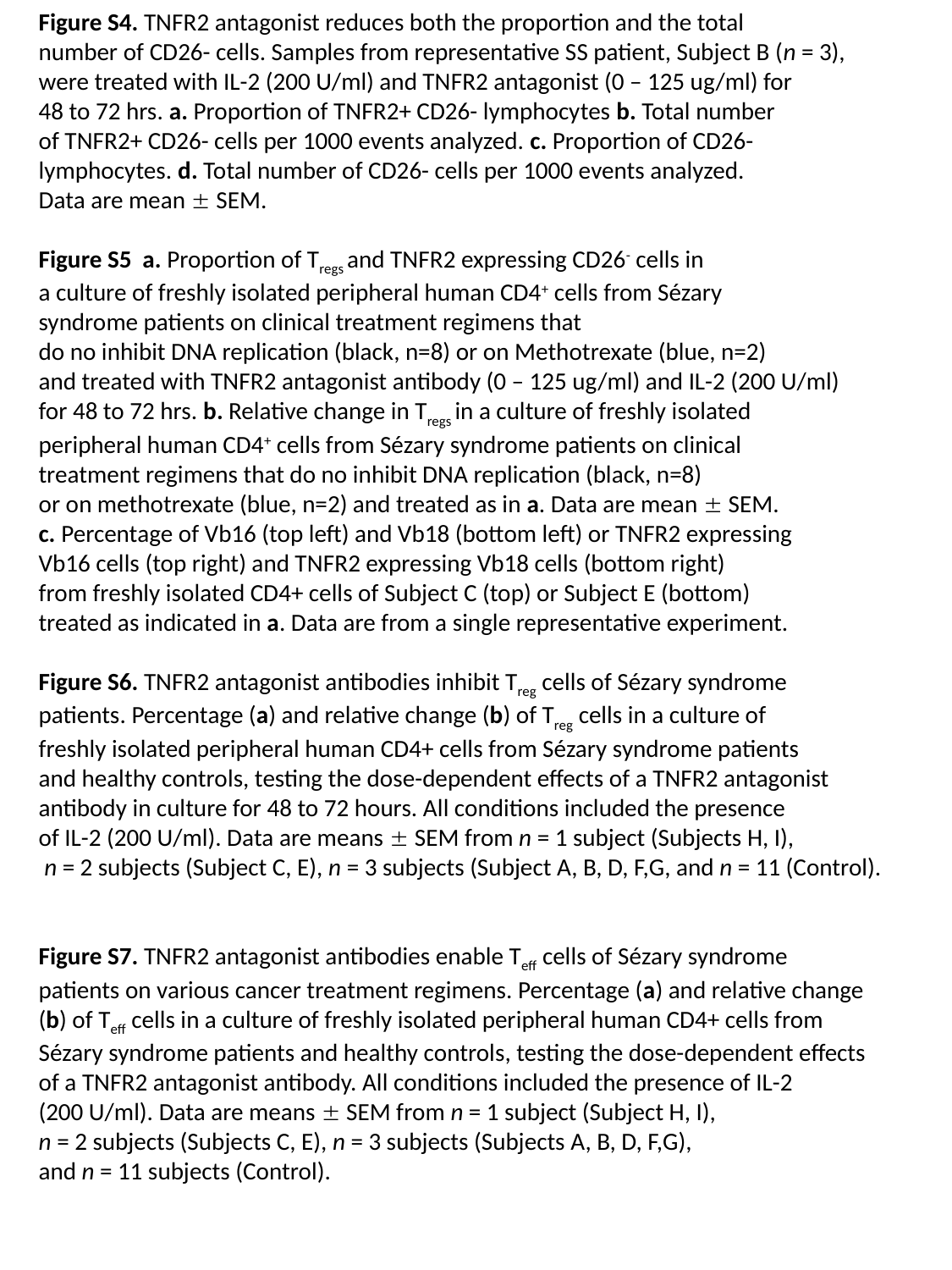

Figure S4. TNFR2 antagonist reduces both the proportion and the total
number of CD26- cells. Samples from representative SS patient, Subject B (n = 3),
were treated with IL-2 (200 U/ml) and TNFR2 antagonist (0 – 125 ug/ml) for
48 to 72 hrs. a. Proportion of TNFR2+ CD26- lymphocytes b. Total number
of TNFR2+ CD26- cells per 1000 events analyzed. c. Proportion of CD26-
lymphocytes. d. Total number of CD26- cells per 1000 events analyzed.
Data are mean ± SEM.
Figure S5 a. Proportion of Tregs and TNFR2 expressing CD26- cells in
a culture of freshly isolated peripheral human CD4+ cells from Sézary
syndrome patients on clinical treatment regimens that
do no inhibit DNA replication (black, n=8) or on Methotrexate (blue, n=2)
and treated with TNFR2 antagonist antibody (0 – 125 ug/ml) and IL-2 (200 U/ml)
for 48 to 72 hrs. b. Relative change in Tregs in a culture of freshly isolated
peripheral human CD4+ cells from Sézary syndrome patients on clinical
treatment regimens that do no inhibit DNA replication (black, n=8)
or on methotrexate (blue, n=2) and treated as in a. Data are mean ± SEM.
c. Percentage of Vb16 (top left) and Vb18 (bottom left) or TNFR2 expressing
Vb16 cells (top right) and TNFR2 expressing Vb18 cells (bottom right)
from freshly isolated CD4+ cells of Subject C (top) or Subject E (bottom)
treated as indicated in a. Data are from a single representative experiment.
Figure S6. TNFR2 antagonist antibodies inhibit Treg cells of Sézary syndrome
patients. Percentage (a) and relative change (b) of Treg cells in a culture of
freshly isolated peripheral human CD4+ cells from Sézary syndrome patients
and healthy controls, testing the dose-dependent effects of a TNFR2 antagonist
antibody in culture for 48 to 72 hours. All conditions included the presence
of IL-2 (200 U/ml). Data are means ± SEM from n­ = 1 subject (Subjects H, I),
 n­ = 2 subjects (Subject C, E), n ­= 3 subjects (Subject A, B, D, F,G, and n­ = 11 (Control).
Figure S7. TNFR2 antagonist antibodies enable Teff cells of Sézary syndrome
patients on various cancer treatment regimens. Percentage (a) and relative change
(b) of Teff cells in a culture of freshly isolated peripheral human CD4+ cells from
Sézary syndrome patients and healthy controls, testing the dose-dependent effects
of a TNFR2 antagonist antibody. All conditions included the presence of IL-2
(200 U/ml). Data are means ± SEM from n­ = 1 subject (Subject H, I),
n­ = 2 subjects (Subjects C, E), n ­= 3 subjects (Subjects A, B, D, F,G),
and n­ = 11 subjects (Control).

## Slide 3
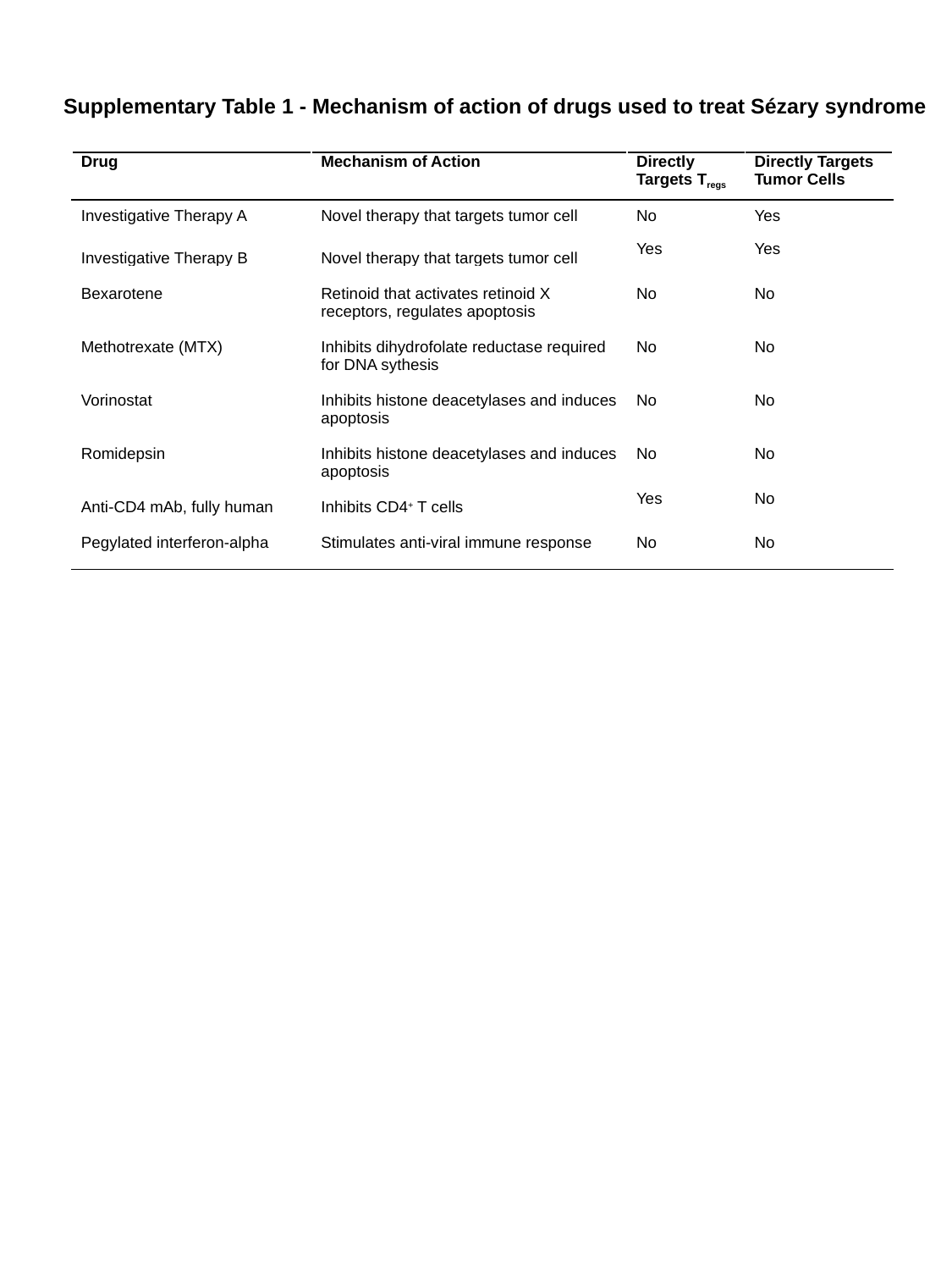

Supplementary Table 1 - Mechanism of action of drugs used to treat Sézary syndrome
| Drug | Mechanism of Action | Directly Targets Tregs | Directly Targets Tumor Cells |
| --- | --- | --- | --- |
| Investigative Therapy A | Novel therapy that targets tumor cell | No | Yes |
| Investigative Therapy B | Novel therapy that targets tumor cell | Yes | Yes |
| Bexarotene | Retinoid that activates retinoid X receptors, regulates apoptosis | No | No |
| Methotrexate (MTX) | Inhibits dihydrofolate reductase required for DNA sythesis | No | No |
| Vorinostat | Inhibits histone deacetylases and induces apoptosis | No | No |
| Romidepsin | Inhibits histone deacetylases and induces apoptosis | No | No |
| Anti-CD4 mAb, fully human | Inhibits CD4+ T cells | Yes | No |
| Pegylated interferon-alpha | Stimulates anti-viral immune response | No | No |

## Slide 4
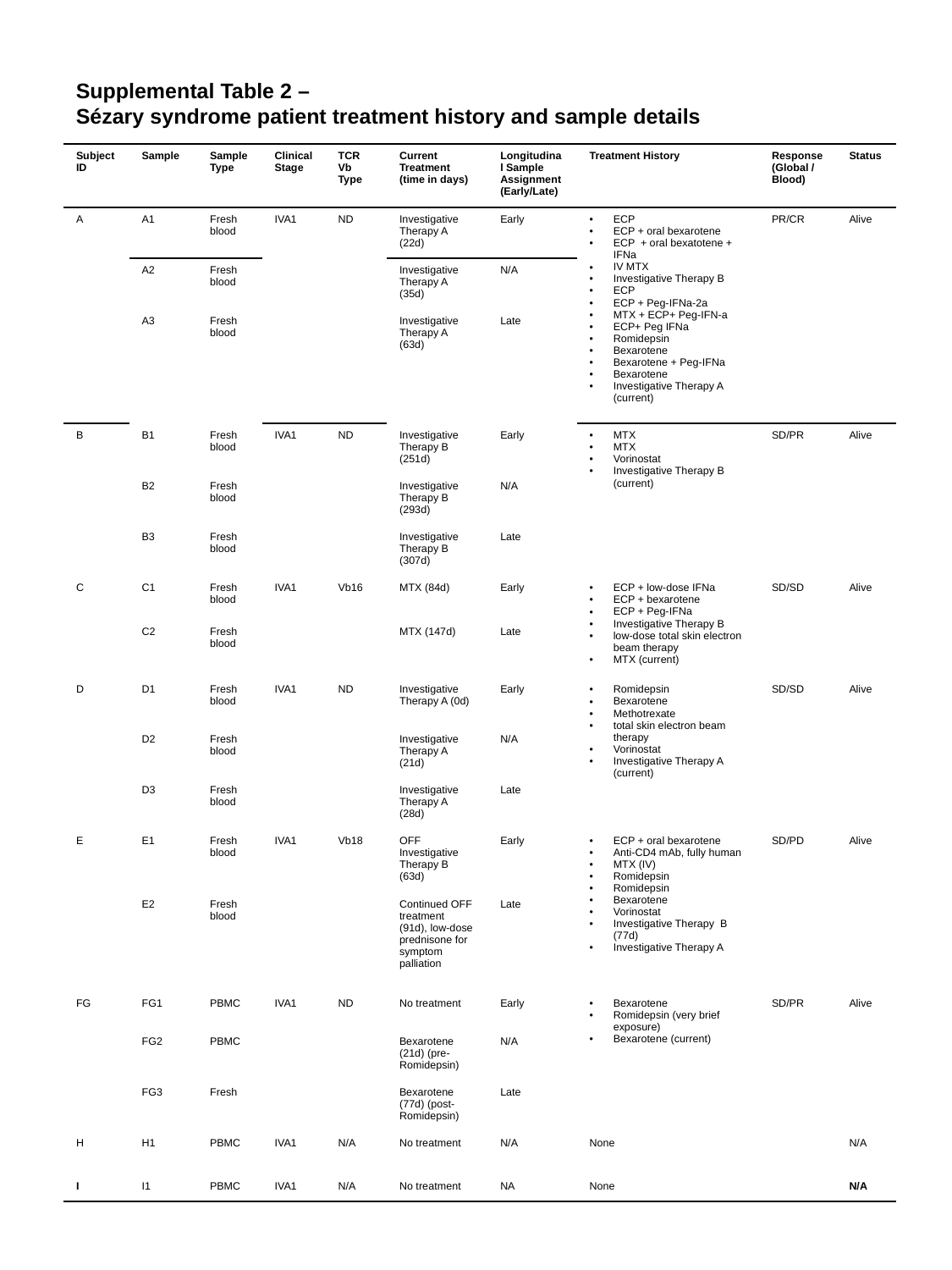

Supplemental Table 2 –
Sézary syndrome patient treatment history and sample details
| Subject ID | Sample | Sample Type | Clinical Stage | TCR Vb Type | Current Treatment (time in days) | Longitudinal Sample Assignment (Early/Late) | Treatment History | Response (Global / Blood) | Status |
| --- | --- | --- | --- | --- | --- | --- | --- | --- | --- |
| A | A1 | Fresh blood | IVA1 | ND | Investigative Therapy A (22d) | Early | ECP ECP + oral bexarotene ECP + oral bexatotene + IFNa IV MTX Investigative Therapy B ECP ECP + Peg-IFNa-2a MTX + ECP+ Peg-IFN-a ECP+ Peg IFNa Romidepsin Bexarotene Bexarotene + Peg-IFNa Bexarotene Investigative Therapy A (current) | PR/CR | Alive |
| | A2 | Fresh blood | | | Investigative Therapy A (35d) | N/A | | | |
| | A3 | Fresh blood | | | Investigative Therapy A (63d) | Late | | | |
| B | B1 | Fresh blood | IVA1 | ND | Investigative Therapy B (251d) | Early | MTX MTX Vorinostat Investigative Therapy B (current) | SD/PR | Alive |
| | B2 | Fresh blood | | | Investigative Therapy B (293d) | N/A | | | |
| | B3 | Fresh blood | | | Investigative Therapy B (307d) | Late | | | |
| C | C1 | Fresh blood | IVA1 | Vb16 | MTX (84d) | Early | ECP + low-dose IFNa ECP + bexarotene ECP + Peg-IFNa Investigative Therapy B low-dose total skin electron beam therapy MTX (current) | SD/SD | Alive |
| | C2 | Fresh blood | | | MTX (147d) | Late | | | |
| D | D1 | Fresh blood | IVA1 | ND | Investigative Therapy A (0d) | Early | Romidepsin Bexarotene Methotrexate total skin electron beam therapy Vorinostat Investigative Therapy A (current) | SD/SD | Alive |
| | D2 | Fresh blood | | | Investigative Therapy A (21d) | N/A | | | |
| | D3 | Fresh blood | | | Investigative Therapy A (28d) | Late | | | |
| E | E1 | Fresh blood | IVA1 | Vb18 | OFF Investigative Therapy B (63d) | Early | ECP + oral bexarotene Anti-CD4 mAb, fully human MTX (IV) Romidepsin Romidepsin Bexarotene  Vorinostat  Investigative Therapy B (77d) Investigative Therapy A | SD/PD | Alive |
| | E2 | Fresh blood | | | Continued OFF treatment (91d), low-dose prednisone for symptom palliation | Late | | | |
| FG | FG1 | PBMC | IVA1 | ND | No treatment | Early | Bexarotene Romidepsin (very brief exposure) Bexarotene (current) | SD/PR | Alive |
| | FG2 | PBMC | | | Bexarotene (21d) (pre-Romidepsin) | N/A | | | |
| | FG3 | Fresh | | | Bexarotene (77d) (post-Romidepsin) | Late | | | |
| H | H1 | PBMC | IVA1 | N/A | No treatment | N/A | None | | N/A |
| I | I1 | PBMC | IVA1 | N/A | No treatment | NA | None | | N/A |

## Slide 5
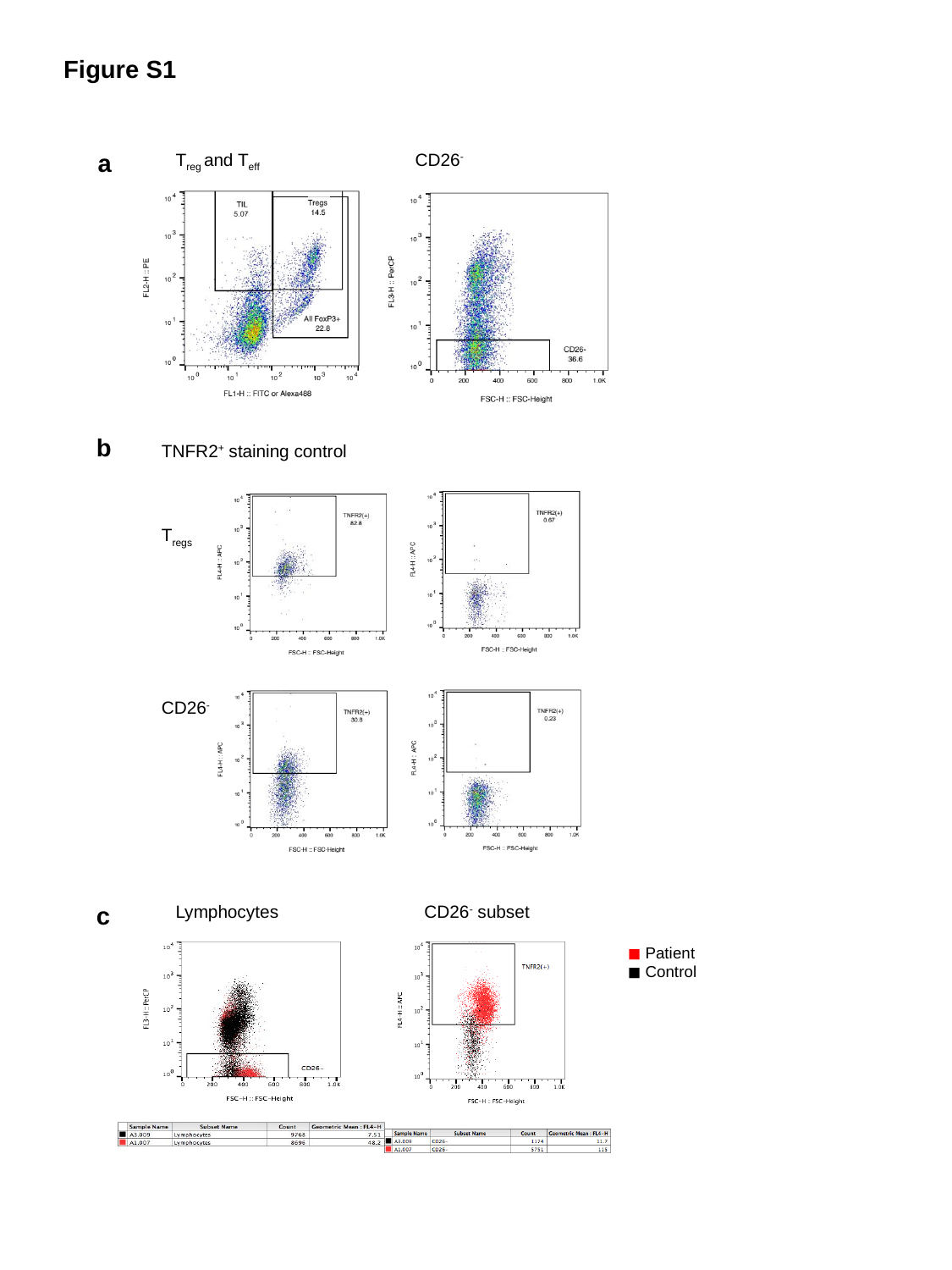

Figure S1
a
Treg and Teff CD26-
b
TNFR2+ staining control
Tregs
CD26-
c
Lymphocytes CD26- subset
◼ Patient
◼ Control

## Slide 6
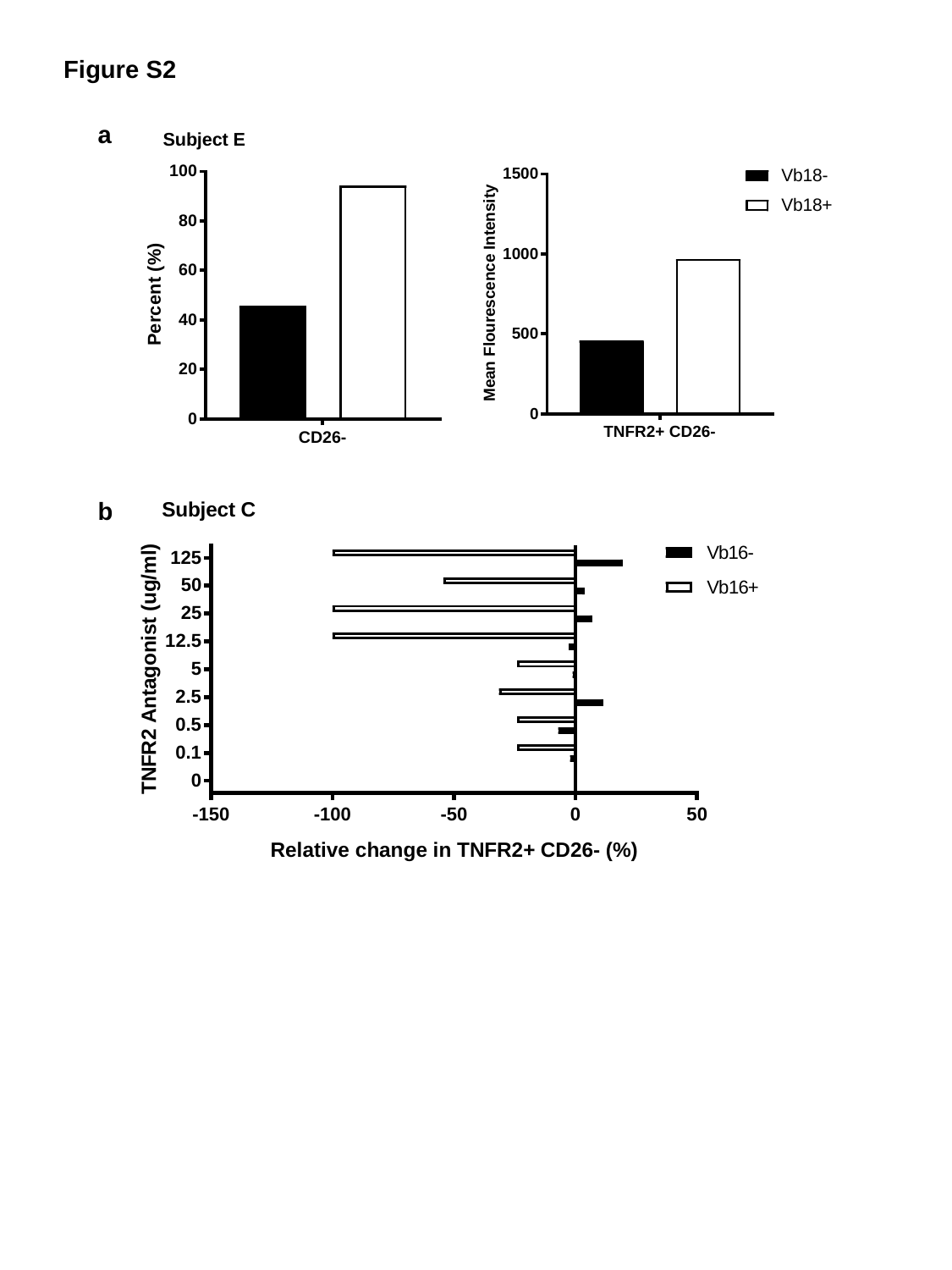

Figure S2
a
b

## Slide 7
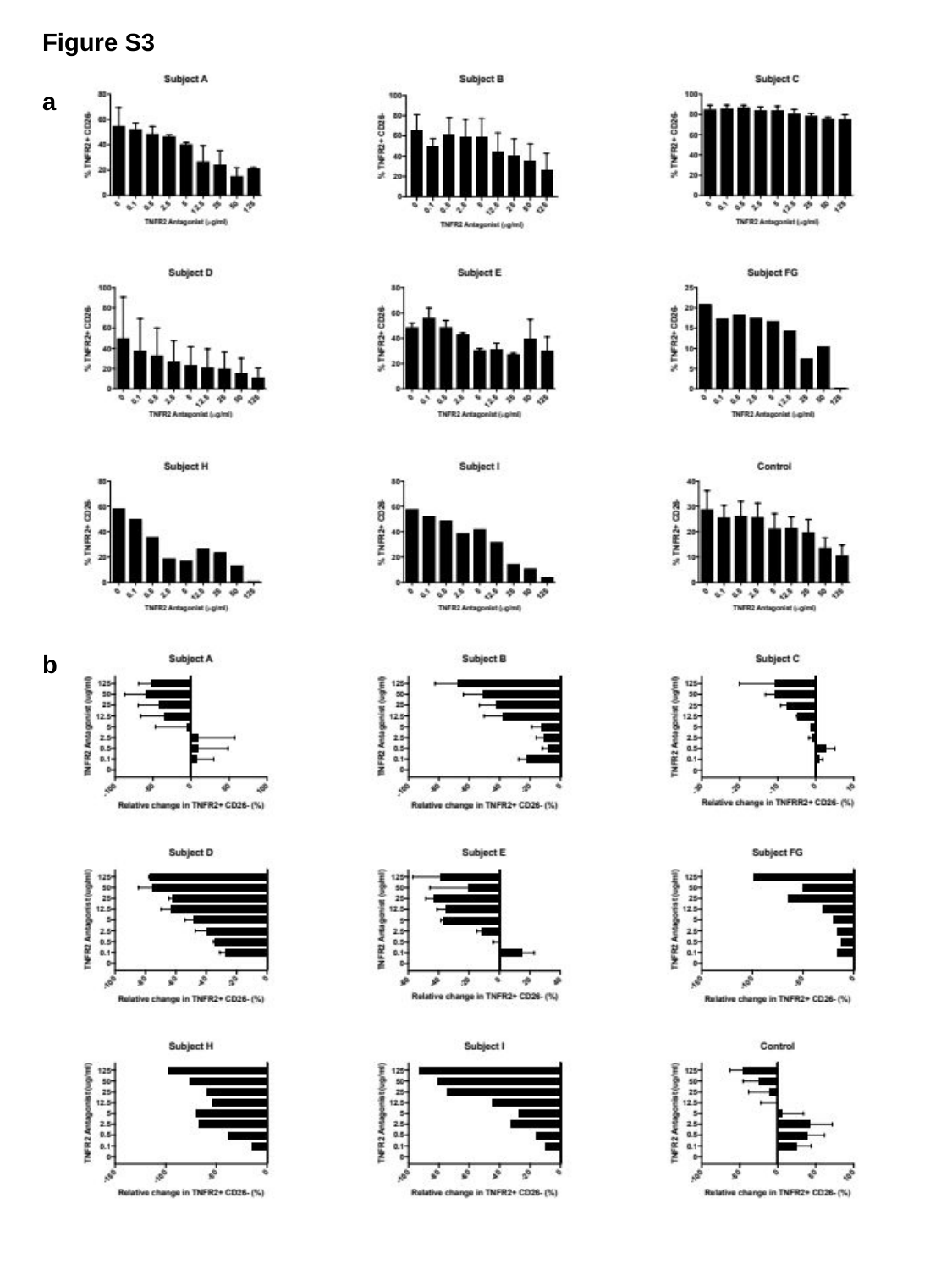

Figure S3
a
b

## Slide 8
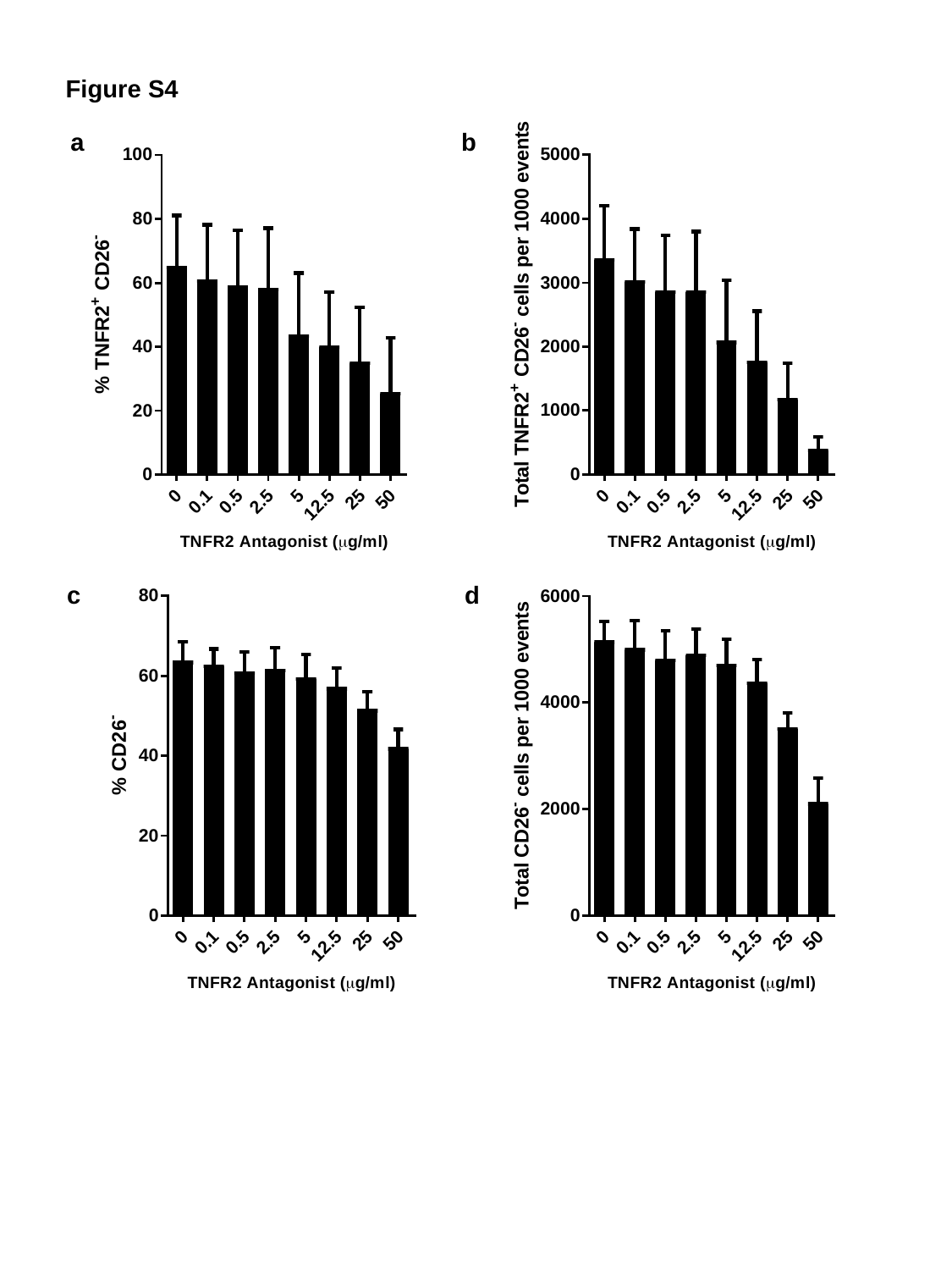

Figure S4
a b
c d

## Slide 9
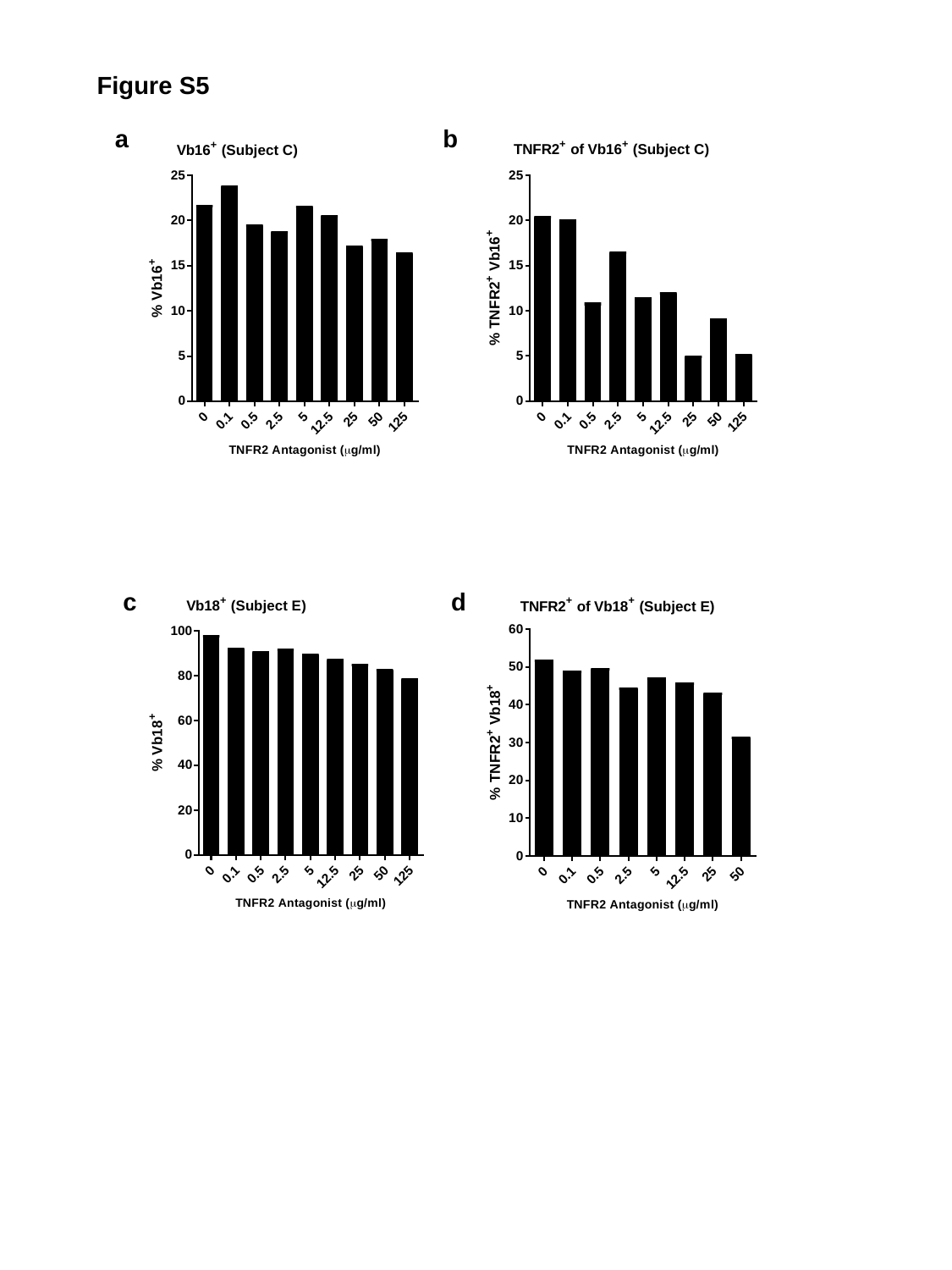

Figure S5
a b
c d

## Slide 10
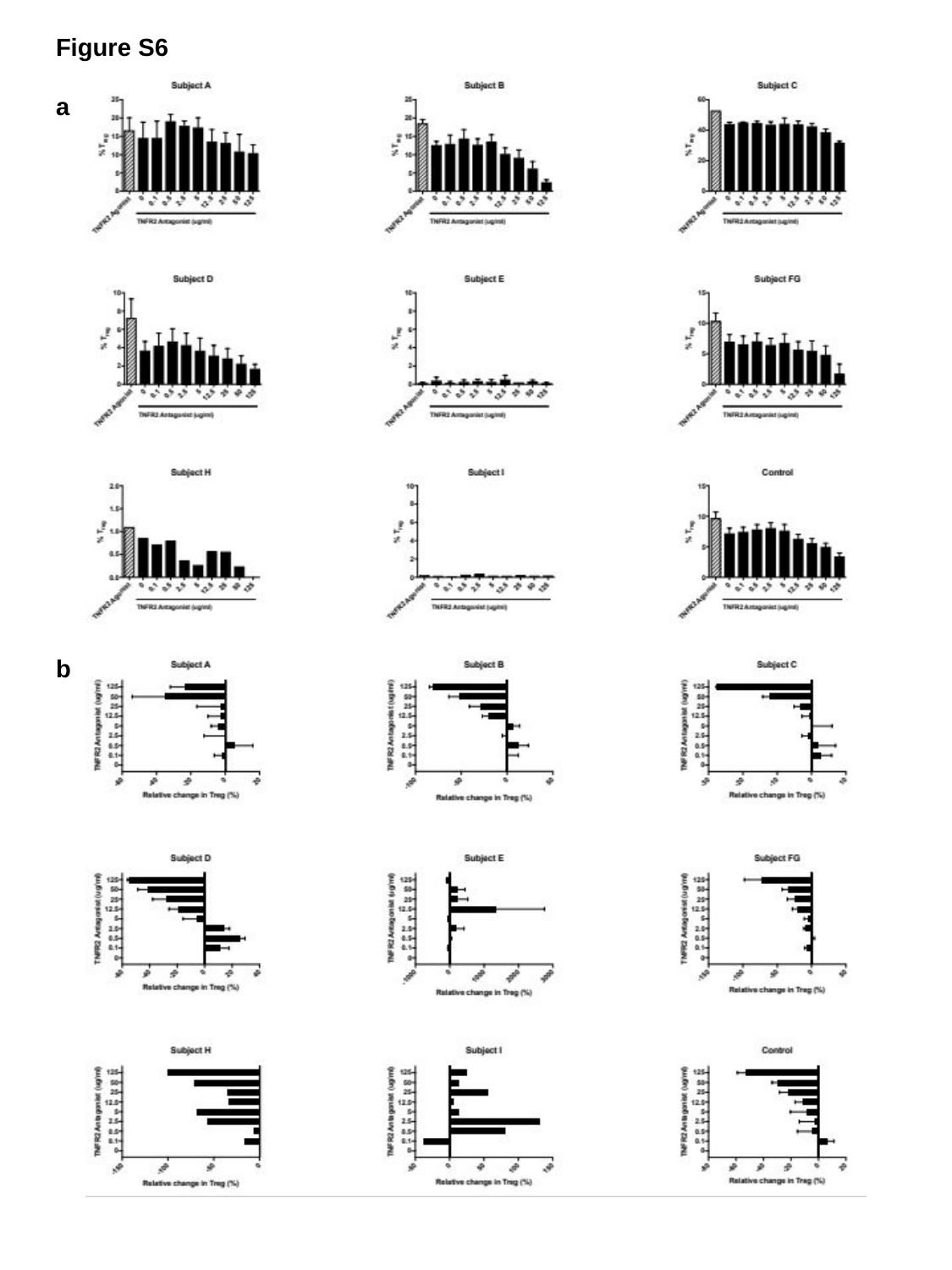

Figure S6
a
b

## Slide 11
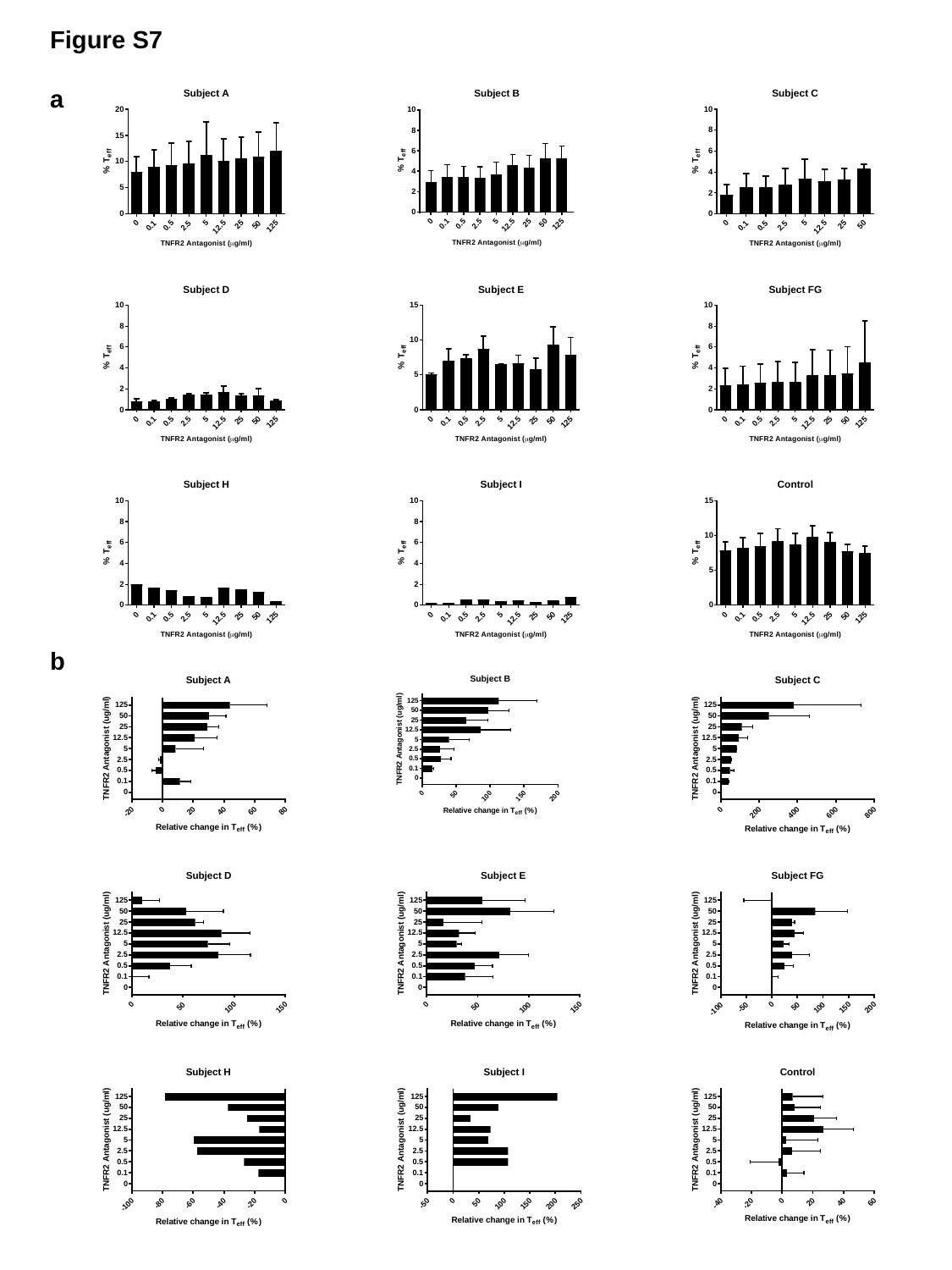

Figure S7
a
b
